# Compostable and Recyclable Baroplastic Triblock Copolymers Enable Low‐Energy Polymer Processing

**DOI:** 10.1002/smll.202514939

**Published:** 2026-03-30

**Authors:** Chengzhang Xu, Chengwei Yi, Emilia Fulajtar, Anja FRM Ramsperger, Julian Brehm, Christian Laforsch, Holger Schmalz, Sabine Rosenfeldt, Ulrich Mansfeld, Holger Kress, Andreas Möglich, Andreas Greiner, Seema Agarwal

**Affiliations:** ^1^ Macromolecular Chemistry and Bavarian Polymer Institute University of Bayreuth Bayreuth Germany; ^2^ Department of Biochemistry University of Bayreuth Bayreuth Germany; ^3^ Animal Ecology I University of Bayreuth Bayreuth Germany; ^4^ Biological Physics University of Bayreuth Bayreuth Germany; ^5^ Physical Chemistry 1 and Bavarian Polymer Institute University of Bayreuth Bayreuth Germany

**Keywords:** baroplastic, biodegradable, block copolymer

## Abstract

Baroplastic polymers enable low‐energy processing at low temperatures under mild pressure preserving polymer integrity and supporting end‐of‐life pathways that reduce the formation of persistent microplastic residues. Poly(*L*‐lactide)‐*block*‐poly(ethylene glycol)‐*block*‐poly(*L*‐lactide) (PLLA‐*b*‐PEG‐*b*‐PLLA) triblock copolymers demonstrate baroplasticity, enabling ambient temperature processing under moderate pressure. Here, we synthesized and characterized PLLA‐*b*‐PEG‐*b*‐PLLA with specific block lengths, showing rapid degradation within 2 months under industrial composting conditions and effective chemical and physical recyclability. Low‐temperature baroplastic processing preserves the activity of encapsulated heat‐sensitive proteins, expanding its application potential. These findings suggest that PLLA‐*b*‐PEG‐*b*‐PLLA combines sustainable processing, compostability, and recyclability, offering a promising platform for environmentally friendly polymer technologies in packaging, agriculture, and beyond.

## Graphical abstract

Baroplastic Poly(*L*‐lactide)‐*block*‐poly(ethylene glycol)‐*block*‐poly(*L*‐lactide) triblock copolymers enable ambient‐temperature processing under moderate pressure, reducing energy demand while preserving polymer integrity. The materials show rapid industrial compostability, effective chemical and physical recyclability, and compatibility with heat‐sensitive proteins, highlighting baroplastic processing as a sustainable platform for packaging, agricultural, and advanced functional polymer applications.

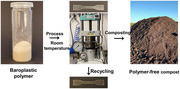

## 1 Introduction

Polymers play a central role in modern society, contributing to countless technological solutions. However, high‐temperature melt processing, the predominant route for thermoplastic materials, is energy‐intensive, limits upcycling, and due to cumulative thermal degradation during processing and reprocessing can ultimately contribute to plastic waste and microplastic formation [1]. A separate challenge arises from the incorporation of temperature‐sensitive additives. Polymers serve as carriers for functional additives in many different fields including packaging, agricultural, pharmaceutical, and medical applications [2, 3, 4, 5]. Conventional approaches for the incorporation of additives, such as solution casting [6], electrospinning [7], or spray drying [8] typically rely on solvents, require long processing times, and suffer from limited scalability [6, 9, 10]. Although melt processing offers an attractive solvent‐free alternative, the high temperatures involved can damage polymers and sensitive additives, thereby restricting its applicability. Beyond processing‐related challenges, most commercial polymers are environmentally persistent and fragment into microplastics that can remain in ecosystems for decades. Compost represents one of the main entry pathways for plastic into the environment [11, 12]. According to a local composting facility (Bio‐Kompost und Entsorgung GmbH & Co. Bayreuth‐Pegnitz KG, personal communication, August 19, 2025), approximately 35 tons of contaminant materials, including significant amounts of plastic, are separated each month from roughly 900 tons of organic waste. However, small‐sized contaminants, including plastic fragments of unknown quantity, cannot be effectively removed and may remain in the compost.

Therefore, sustainable alternatives to conventional plastics are urgently needed. In this context, recyclable polymer materials are essential for conserving resources and enabling circular material use, while compostability and biodegradability in compost, represent an important additional advantage for applications with a higher likelihood of entering composting streams, such as packaging [13]. Moreover, application areas such as agriculture, cosmetics, personal care, and laundry products could also particularly benefit from biodegradable polymers, as materials used in these sectors are often difficult or economically unfeasible to collect for recycling [14, 15].

Baroplastic polymers provide sustainable alternatives to conventional thermoplastics. Unlike their traditional counterparts, they can be shaped at low temperatures under moderate pressure, dramatically reducing energy demand [16, 17, 18, 19]. This pressure‐induced processing is mechanistically distinct from thermal sintering and has been demonstrated in block copolymers such as polystyrene‐*block*‐poly(*n*‐butyl methacrylate), blends, and core–shell particles [20, 21, 22, 23]. In baroplastic materials, pressure triggers an order–disorder transition of the block copolymer / blend morphology with low (soft component) and high glass transition temperatures (*T*_g_s) (hard component), resulting in a change from a structurally ordered solid to a disordered, melt‐like state that enables flow and shaping. Baroplastic polymers with potential biodegradability can provide a sustainable polymer platform that simultaneously addresses processing challenges and the environmental persistence of conventional polymers in specific application contexts.

Taniguchi and Lovell demonstrated in 2012 the baroplastic behavior of diblock copolymers based on poly(*L*‐lactide) (PLLA) as the hard segment combined with soft segments such as poly(1,5‐dioxepan‐2‐one) (PDXO), an amorphous polymer with a *T*_g_ between −36 and −32°C, and poly(*ε*‐caprolactone‐*r*‐5‐ethylene ketal *ε*‐caprolactone), exhibiting *T*_g_ values between −58 and −48°C [24]. Baroplastic behavior was observed for specific block copolymer compositions containing 40–60 wt.% PLLA. In contrast, when the soft segment was a semicrystalline low‐*T*_g_ polymer, such as poly(*ε*‐caprolactone), baroplasticity was not observed at room temperature but only at temperatures above the melting point of the soft segment. More than a decade after their initial report, the degradability of PLLA–*b*‐PDXO diblock copolymers has primarily been investigated through enzymatic degradation studies using lipase PS and proteinase K [25]. While such experiments conducted under controlled laboratory conditions provide insight into specific degradation mechanisms, they do not necessarily reflect the actual biodegradability of polymers in complex environmental systems. To date, there is limited evidence for the effective environmental degradation of these so‐called biodegradable block copolymers. For example, soil burial experiments have reported weight losses of less than 3 wt.% after 4 weeks in soil [19].

More generally, aliphatic polyesters such as PLLA are widely classified in the literature as biodegradable polymers. However, substantial evidence suggests that their degradation can be very slow under realistic environmental conditions, including industrial composting, wastewater, and soil environments [26, 27, 28]. As a result, the widespread use of such materials as “biodegradable” polymers may unintentionally exacerbate environmental pollution if polymer fragments persist in the environment for extended periods rather than undergoing complete bioassimilation to carbondioxide, water, and biomass. Consequently, baroplastic polymer systems that combine low‐energy processability with well‐defined and sufficiently rapid degradation without any microplastic formation under relevant environmental conditions remain scarce and warrant systematic investigation. Establishing libraries of such materials with defined processing windows, mechanical properties, and environmental degradation profiles would enable the rational selection of polymers tailored to specific applications, where both performance and end‐of‐life behavior are critical.

**FIGURE 1:**
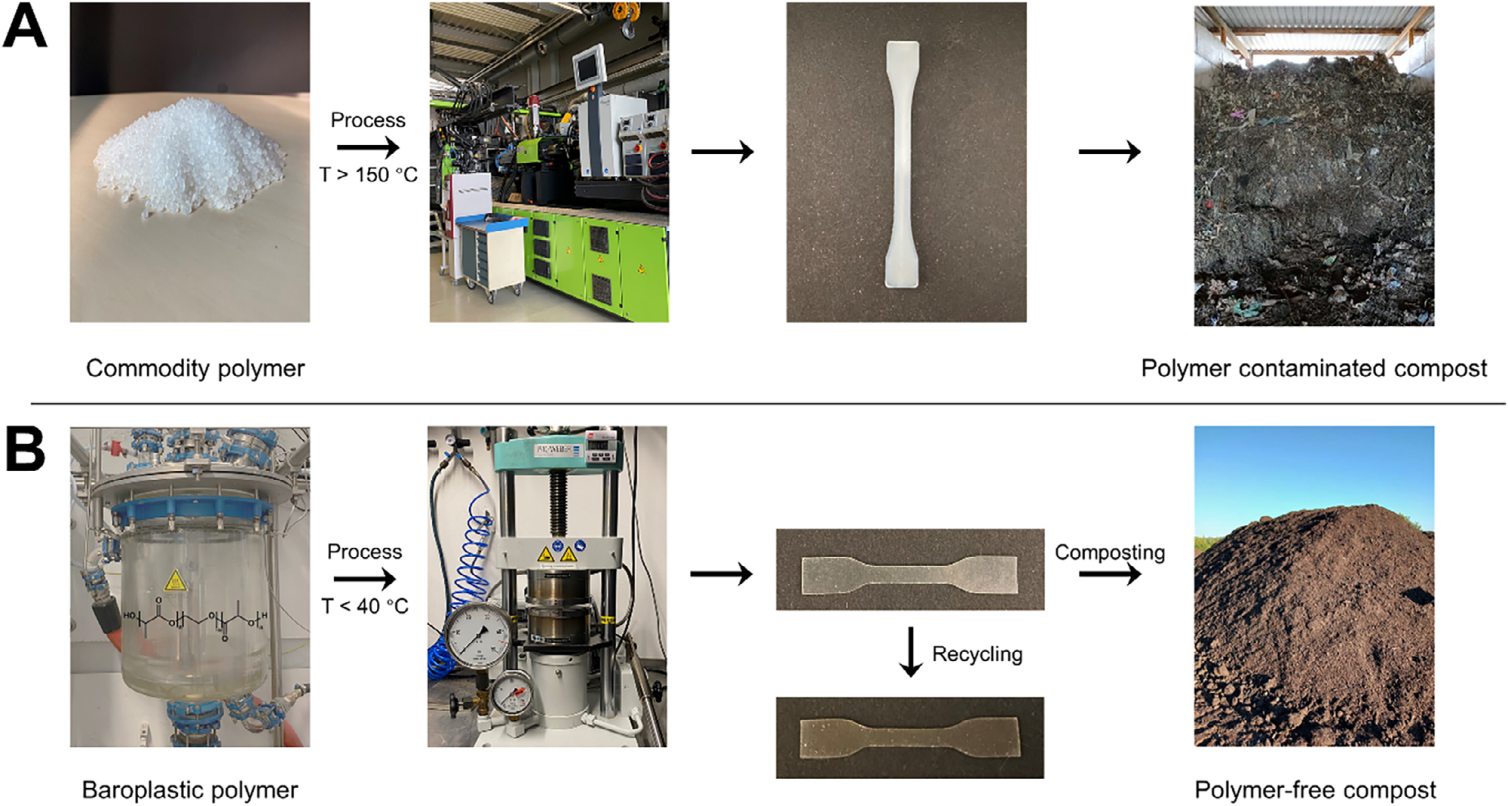
Typical life cycle of polymers. (A) Life cycle of a conventional commodity plastic disposed improperly leading to contaminated compost. Polymer pellets are thermally processed (e.g., injection molding at high temperatures). After use, the product is discarded in landfills and contributes to microplastic contamination in compost, requiring additional separation efforts. (B) Life cycle of a compostable and recyclable baroplastic polymer leading to polymer‐free compost even if disposed improperly. The synthesized baroplastic is processed at moderate temperatures under pressure. After use, it can be recycled (physically or chemically) or composted, leaving no microplastic residues as evidenced by biodegradation tests monitored by respirometry and extraction.

Here, we report that PLLA‐*block*‐poly(ethylene glycol)‐*block*‐PLLA (PLLA‐*b*‐PEG‐*b*‐PLLA) triblock copolymers with semicrystalline segments of certain compositions exhibit remarkable processability at low temperature under elevated pressure, far below their melting points. To the best of our knowledge, such behavior has not been previously reported for PLLA‐*b*‐PEG‐*b*‐PLLA. It contains hydrophilic soft PEG segments and hard PLLA segments. Temperature sensitive additives, like proteins, could also be encapsulated under mild baroplastic conditions. Importantly, we analyze the compostability of PLLA‐*b*‐PEG‐*b*‐PLLA and demonstrate that it undergoes rapid degradation under standard composting conditions and thereby minimizing microplastic accumulation, an aspect likewise not previously reported to our knowledge for baroplastics. In addition, the polymer allows both chemical and mechanical recycling, providing a circular life cycle (Figure 1). Ecotoxicological assessments confirm its compatibility with model organisms, highlighting its environmental safety. PLLA‐*b*‐PEG‐*b*‐PLLA belongs to a well‐established, commercially accessible polymer class, facilitating scalable and sustainable production. This combination of straightforward synthetic accessibility, low‐temperature baroplastic processability, compostability, recyclability, and environmental compatibility positions PLLA‐*b*‐PEG‐*b*‐PLLA block copolymers as a versatile platform for sustainable polymer technologies, enabling applications across packaging, and other fields while contributing to a circular materials economy.

## 2 Results and Discussion

### 2.1 Polymer Synthesis and Characterization

Block copolymers were synthesized by ring‐opening polymerization of lactide (*L*‐ and *D*,*L*‐lactide) in the presence of chain‐end hydroxy‐functionalized PEG and Sn(Oct)_2_ following a standard protocol. A series of A‐B and A‐B‐A type block copolymers with varied PLA chain lengths was obtained to determine the structural requirements and processing conditions for baroplasticity, i.e., processing via plastic deformation under moderate pressure (ca. 10 MPa) and ambient temperature. For the synthesis of diblock copolymers, PEG with only one chain‐end functionalized with a hydroxy group was used. The other chain‐end was methoxy‐terminated (mPEG). Structural characterization and compositional analysis of block copolymers were performed using proton nuclear magnetic resonance (^1^H NMR) spectroscopy. The characteristic peaks for the PLLA (1.6, 5.1 ppm) and the PEG (3.6 ppm) blocks were clearly identified in ^1^H NMRin CDCl_3_ and the peak integration of methine protons of PLLA at 5.1 ppm and methylene protons of PEG at 3.6 ppm were used for the copolymer composition determination (Table 1; Figure S1 and Equations S1–S3). The block copolymers are designated with the abbreviation of the respective segments and a number in the subscript that shows the average number of repeat units of that particular segment. For example, a triblock copolymer of PLLA and PEG designated as PLLA_93_‐*b*‐PEG_200_‐*b*‐PLLA_93_ has PLLA segments with 93 repeat units each and PEG with 200 repeat units. The ^1^H NMR spectra of all diblock and triblock copolymers are shown in Figures S2–S5. Molecular weights were calculated from ^1^H NMR and experimentally determined by gel permeation chromatography (GPC) (Figures S1–S6; Table 1). The GPC traces of all block copolymers were unimodal with relatively low dispersity. The number‐average molecular weights determined by GPC were consistently higher than those obtained from ^1^H NMR spectroscopy. This discrepancy arises from the fundamentally different principles of the two methods: GPC provides relative molecular weights based on hydrodynamic volume and calibration with polystyrene standards, whereas NMR yields absolute M_n_ values derived from end‐group analysis. For amphiphilic PLLA‐based block copolymers, differences in chain conformation and solvent interactions relative to polystyrene lead to systematic overestimation of M_n_ by GPC.

**TABLE 1:** Molecular characterization of the synthesized block copolymers.

Sample	Polymer	Yield/ %	EG:LA (in feed, molar ratio)	EG:LA (from ^1^H NMR, molar ratio)	${\bar{M}}_{n}$ (^1^H NMR)	${\bar{M}}_{n}$ (GPC)	*Đ*
1	mPEG_120_‐*b*‐PDLLA_49_	78	120:95	120:49	8300	16 600	1.17
2	mPEG_120_‐*b*‐PDLLA_142_	82	120:159	120:142	14 600	33 500	1.36
3	mPEG_120_‐*b*‐PDLLA_188_	81	120:212	120:188	17 800	32 400	1.33
4	mPEG_120_‐*b*‐PDLLA_308_	88	120:317	120:308	25 900	32 600	1.57
5	mPEG_120_‐*b*‐PDLLA_406_	88	120:423	120:406	32 600	47 900	1.68
6	mPEG_120_‐*b*‐PLLA_157_	91	120:159	120:157	15 600	24 500	1.30
7	mPEG_120_‐*b*‐PLLA_261_	89	120:265	120:261	22 800	29 600	1.52
8	PDLLA_15_‐*b*‐PEG_200_‐*b*‐PDLLA_15_	79	200:110	200:30	10 000	34 200	1.25
9	PDLLA_54_‐*b*‐PEG_200_‐*b*‐PDLLA_54_	61	200:198	200:108	15 000	38 800	1.14
10	PDLLA_110_‐*b*‐PEG_200_‐*b*‐PDLLA_110_	94	200:248	200:220	22 400	35 600	1.24
11	PDLLA_129_‐*b*‐PEG_200_‐*b*‐PDLLA_129_	86	200:330	200:258	24 800	52 500	1.16
12	PDLLA_200_‐*b*‐PEG_200_‐*b*‐PDLLA_200_	78	200:440	200:400	34 200	35 900	1.45
13	PDLLA_259_‐*b*‐PEG_200_‐*b*‐PDLLA_259_	81	200:549	200:518	42 000	31 300	1.86
14	PDLLA_319_‐*b*‐PEG_200_‐*b*‐PDLLA_319_	88	200:659	200:638	49 900	73 500	1.92
15	PLLA_31_‐*b*‐PEG_200_‐*b*‐PLLA_31_	75	200:110	200:62	12 000	23 200	1.09
16	PLLA_64_‐*b*‐PEG_200_‐*b*‐PLLA_64_	83	200:141	200:128	16 300	29 800	1.09
17	PLLA_70_‐*b*‐PEG_200_‐*b*‐PLLA_70_	78	200:198	200:140	17 200	28 300	1.12
18	PLLA_75_‐*b*‐PEG_200_‐*b*‐PLLA_75_	76	200:198	200:150	17 800	30 800	1.10
19	PLLA_78_‐*b*‐PEG_200_‐*b*‐PLLA_78_	79	200:209	200:156	18 200	29 700	1.12
20	PLLA_93_‐*b*‐PEG_200_‐*b*‐PLLA_93_	96	200:198	200:186	20 200	34 400	1.12
21	PLLA_118_‐*b*‐PEG_200_‐*b*‐PLLA_118_	96	200:248	200:236	23 400	45 700	1.14
22	PLLA_200_‐*b*‐PEG_200_‐*b*‐PLLA_200_	91	200:440	200:400	34 200	76 300	1.09
23	PLLA_35_‐*b*‐PEG_455_‐*b*‐PLLA_35_	87	455:100	455:70	25 000	44 900	1.09
24	PLLA	88	—	—	—	10 600	1.90

The thermal transition temperatures as measured by differential scanning calorimetry (DSC) are listed in Table S1. All block copolymers exhibited a melting transition corresponding to the PEG block. Depending on the nature of the second block, amorphous PDLLA (starting monomer *D,L*‐lactide) or semicrystalline PLLA (starting monomer *L*‐lactide), a second melting peak was observed in the case of PLLA (Figures S7–S9).

**FIGURE 2:**
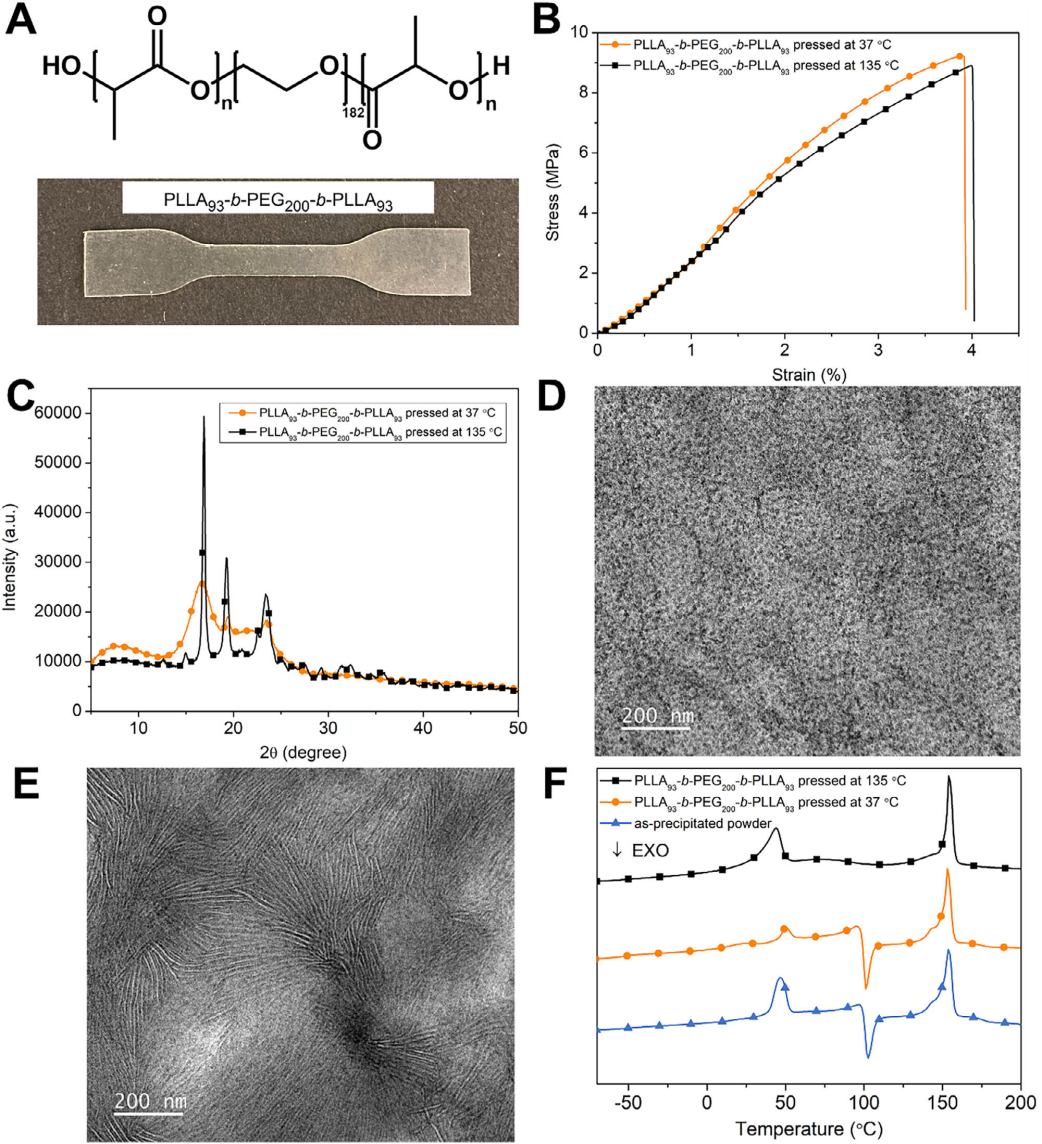
Structural analysis of PLLA_93_‐*b*‐PEG_200_‐*b*‐PLLA_93_ pressed at different temperatures. (A) General chemical formula of the PLLA_n_‐*b*‐PEG_200_‐*b*‐PLLA_n_ triblock copolymers (indices denote the average degree of polymerization of the respective blocks) and photograph of a dogbone sample of PLLA_93_‐*b*‐PEG_200_‐*b*‐PLLA_93_ pressed at 37°C/10 MPa/5 min (sample 20, Table S1, sample thickness 500 µm). (B) Comparison of stress vs. strain behavior and (C) XRD patterns of PLLA_93_‐*b*‐PEG_200_‐*b*‐PLLA_93_ (sample 20) processed as dogbone shape films at different temperatures (orange dots and line: pressed at 37°C/10 MPa/14 h, and black squares and line: pressed at 135°C/10 MPa/5 min). (D) TEM micrographs of RuO_4_‐stained thin sections of PLLA_93_‐*b*‐PEG_200_‐*b*‐PLLA_93_ sample pressed at 37°C/10 MPa/14 h. (E) TEM micrographs of RuO_4_‐stained thin sections of PLLA_93_‐*b*‐PEG_200_‐*b*‐PLLA_93_ sample pressed at 135°C/10 MPa/5 min. (F) Comparison of DSC traces (heating rate 10 K/min under nitrogen atmosphere, first heating run) of PLLA_93_‐*b*‐PEG_200_‐*b*‐PLLA_93_ pressed samples obtained under different conditions (blue triangles and line: as‐precipitated powder, orange dots and line: pressed at 37°C/10 MPa/14 h and black squares and line: pressed at 135°C/10 MPa/5 min).

The synthesized block copolymers were tested for plasticity at low temperature under pressure by compression molding at 37°C under 10 MPa. Among all block copolymers, only the PLLA‐*b*‐PEG‐*b*‐PLLA triblock copolymers with specific PLLA block lengths (PLLA_75_‐*b*‐PEG_200_‐*b*‐PLLA_75_, PLLA_78_‐*b*‐PEG_200_‐*b*‐PLLA_78_, PLLA_93_‐*b*‐PEG_200_‐*b*‐PLLA_93_, PLLA_118_‐*b*‐PEG_200_‐*b*‐PLLA_118_; samples 18–21 in Table 1) were processable at low‐temperature, yielding mechanically stable films within 5 min under pressure (Figure 2A). These polymers can also be processed at lower temperatures (22°C) and at lower pressures (5 MPa). However, 37°C/10 MPa was chosen for subsequent baroplastic processing to ensure consistency. In contrast, other block copolymers of this series occasionally formed films under baroplastic conditions, but these films were fragile and broke upon handling (Figure S10). The observation that only a subset of the triblock copolymers exhibits baroplastic behavior is tentatively attributed to the specific interplay between block architecture and segmental order. Block copolymers of PDLLA with PEG did not exhibit baroplastic behavior under the investigated conditions. This lack of baroplasticity is likely related to the amorphous nature with low *T*_g_ of PDLLA, which results in a uniformly soft material rather than a distinct segregation into hard and soft segments. The lack of distinct segregation into hard and soft segments in diblock copolymers of PLLA with PEG in the composition range studied in the present work might also be the reason for the lack of baroplasticity. Baroplasticity relies on the presence of mechanically robust domains that undergo a pressure‐induced order–disorder transition; in the absence of sufficiently rigid hard segments, such pressure‐responsive morphological transitions are suppressed. Within the series of triblock copolymers of PLLA with PEG, baroplasticity is observed only for PLLA‐*b*‐PEG_200_‐*b*‐PLLA triblock copolymers with PLLA block lengths of ≈75–118 repeat units, indicating a specific composition window in which sufficiently strong PLLA domains coexist with enough soft PEG to enable pressure‐induced flow; shorter PLLA blocks or longer PEG blocks fall outside this window and do not show baroplasticity.

To benchmark against conventional processing, the baroplastically processable copolymers were also hot‐pressed at 135°C/10 MPa/5 min. Their mechanical properties were compared to those of films obtained under baroplastic conditions. As shown for PLLA_93_‐*b*‐PEG_200_‐*b*‐PLLA_93_ (Figure 2B), both processing routes yielded films with comparable strength and toughness, confirming that pressure‐induced flow (baroplasticity) enables low‐temperature processability.

Phase separation of the segments in block copolymers showing baroplasticity was obvious from small‐angle X‐ray scattering (SAXS, Figures S11–S13) with significantly smaller PEG phases in the film pressed at 37°C compared to the film pressed at 135°C. Further, transmission electron microscopy (TEM) showed that samples processed under baroplastic conditions contained irregular crystallites, whereas those processed at high temperature exhibited regular crystallite formation (Figure 2D,E; Figure S14). The results from TEM are in line with the results from XRD measurements (Figure 2C), showing broader peaks corresponding to smaller crystallite sizes due to spatial confinement for the sample pressed at 37°C with its nanophase domains compared to the sharp diffraction peaks for the sample pressed at 135°C with its larger lamellar semicrystalline phases. This observation is supported quantitatively by comparing the heat of fusion (*ΔHf*) of the PEG block: 34 J/g for the hot‐pressed sample (135°C), 25 J/g for the baroplastically pressed sample (37°C), and 18 J/g for the pristine triblock copolymer prior to any processing step (as‐precipitated sample). The first heating thermograms for the baroplastically pressed and as‐precipitated samples exhibited prominent cold crystallization exotherms at approximately 100°C, corresponding to initial crystalline fractions of 31% and 32%, respectively (Figure 2F). In contrast, the hot‐pressed sample—subjected to immediate water quenching—exhibited a negligible exotherm but reached a final crystallinity of only 30%, the lowest among the three processing methods. While the hot‐pressed sample reached a more stable state (evidenced by the lack of further crystallization upon heating), the rapid cooling rate effectively suppressed the overall extent of PLLA crystallization compared to the strain‐induced or precipitation‐driven mechanisms. Finally, the first heating endotherm observed near 50°C in all samples can be attributed to the melting of the PEG block.

### 2.2 Environmental Impacts

**FIGURE 3:**
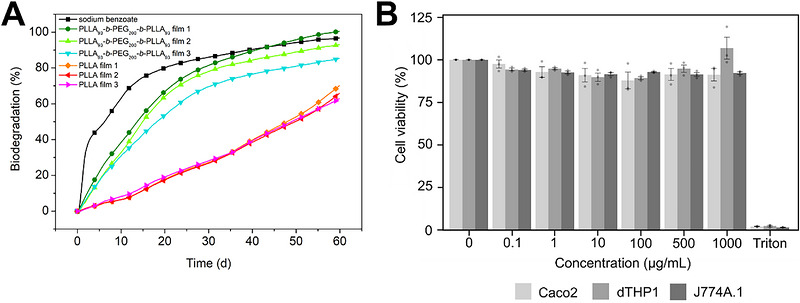
Environmental impact of baroplastic. (A) Biodegradation displayed as percentage of sodium benzoate (positive control, black square line), PLLA_93_‐*b*‐PEG_200_‐*b*‐PLLA_93_ film, triplicate (green dot line, light green up‐triangle line, cyan down‐triangle line), and PLLA film (orange diamond line, pink right‐triangle line, red left‐triangle line). (B) Cell toxicity test with suspension of PLLA_118_‐*b*‐PEG_200_‐*b*‐PLLA_118_ (sample 21). Data represent mean ± SD of *n* = 3 independent measurements.

In our preliminary experiments, the potential environmental impact of the baroplastic polymers was assessed through biodegradation experiments in compost complemented by compatibility tests with cells and aquatic organisms. In compost, PLLA_93_‐*b*‐PEG_200_‐*b*‐PLLA_93_ films degraded significantly faster than PLLA, a commonly used biodegradable reference material, as evidenced by the higher CO_2_ evolution (Figure 3A) [29]. Although the degradation rate slowed after approximately 30 days, active biodegradation was still evident up to 60 days, after which the test was stopped, as indicated by the respirometric profile in Figure 3A and Figure S15. These results are promising, as industrial composting processes typically operate over significantly longer time periods, suggesting the potential for further degradation under practical composting conditions. The design concept of the present baroplastic polymers provides an additional advantage, as the PEG segments are also susceptible to microbial degradation, thereby avoiding the formation of environmentally persistent residues [30, 31, 32].

Environmental compatibility of the baroplastic polymers was further assessed in suspension using mammalian cell lines (the murine macrophage cell line J774A.1, the human adenocarcinoma epithelial cell line Caco2, and human macrophages derived from the differentiation of monocytic cells dTHP‐1) and *Daphnia magna* as a reference organism [33]. In all cases, the baroplastic suspensions exhibited no evidence of severe cytotoxicity or acute aquatic toxicity, indicating principal compatibility with both mammalian and aquatic organisms (Figure 3B, detail given in Supplementary Information).

### 2.3 Physical and Chemical Recycling

**FIGURE 4:**
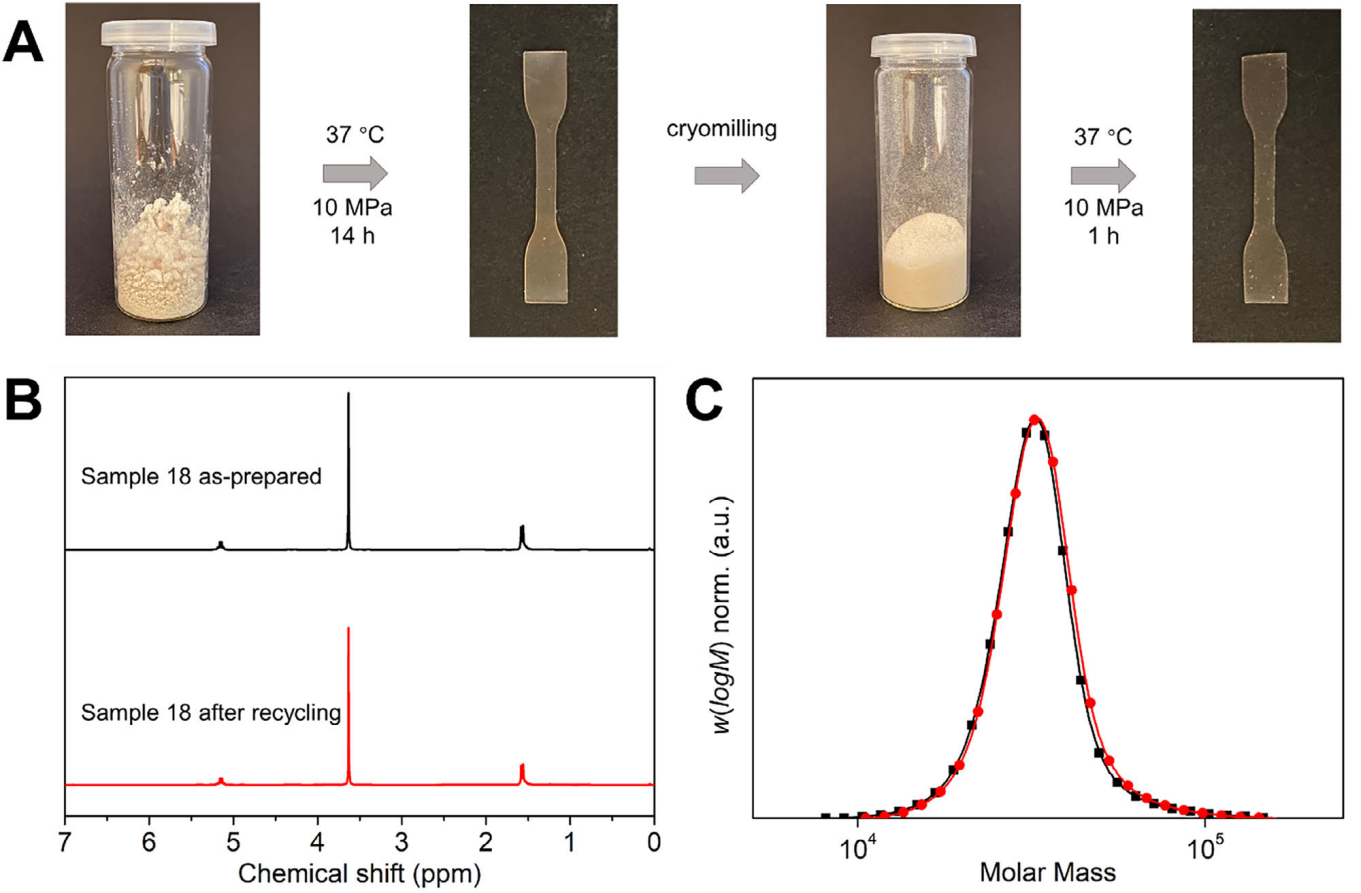
Recycling and characterization of PLLA_93_‐*b*‐PEG_200_‐*b*‐PLLA_93_ (sample 20). (A) Photographs of the as‐prepared polymer, a film pressed at 37°C under 10 MPa for 14 h, the cryo‐milled powder of this film (30 s milling), and the recycled film obtained by baroplastic pressing the cryo‐milled powder at 37°C under 10 MPa for 1 h. Films were cut into dogbone specimens using a hydraulic punch. Characterization of the recycled sample: ^1^H NMR spectra of the recycled baroplastic compared with the as‐prepared sample (B), and molar mass distributions determined by GPC for the as‐prepared sample (black squares) and the recycled film (red dots) (C).

The physical and chemical recycling of compostable baroplastic polymers was investigated to probe their potential for a circular life cycle. Our compostable baroplastic PLLA‐*b*‐PEG‐*b*‐PLLA enables physical recycling at ambient temperature. We demonstrated this by cryo‐milling and re‐pressing of PLLA_93_‐*b*‐PEG_200_‐*b*‐PLLA_93_ (sample 20, Table S1) films (Figure 4A). ^1^H NMR and GPC analyses confirmed that the recycled films retained their molecular characteristics without detectable degradation (Figure 4B,C). Chemical recycling was investigated by the hydrolytic degradation of the PLLA_93_‐*b*‐PEG_200_‐*b*‐PLLA_93_ film using NaOH at 37°C. The degradation products were recovered via solvent extraction using chloroform and diethyl ether and found to be lactic acid and PEG in quantitative yields by ^1^H NMR (Figure S16). After degradation, PEG was recovered from both the chloroform and ether phases, whereas lactic acid was recovered from the chloroform phase. This observation demonstrates efficient depolymerization and selective recovery of the individual components from the copolymer. Although no chain ends could be identified by NMR spectroscopy, the polymer structure suggests the recovery of hydroxy‐end‐capped PEG.

### 2.4 Protein Encapsulation

**FIGURE 5:**
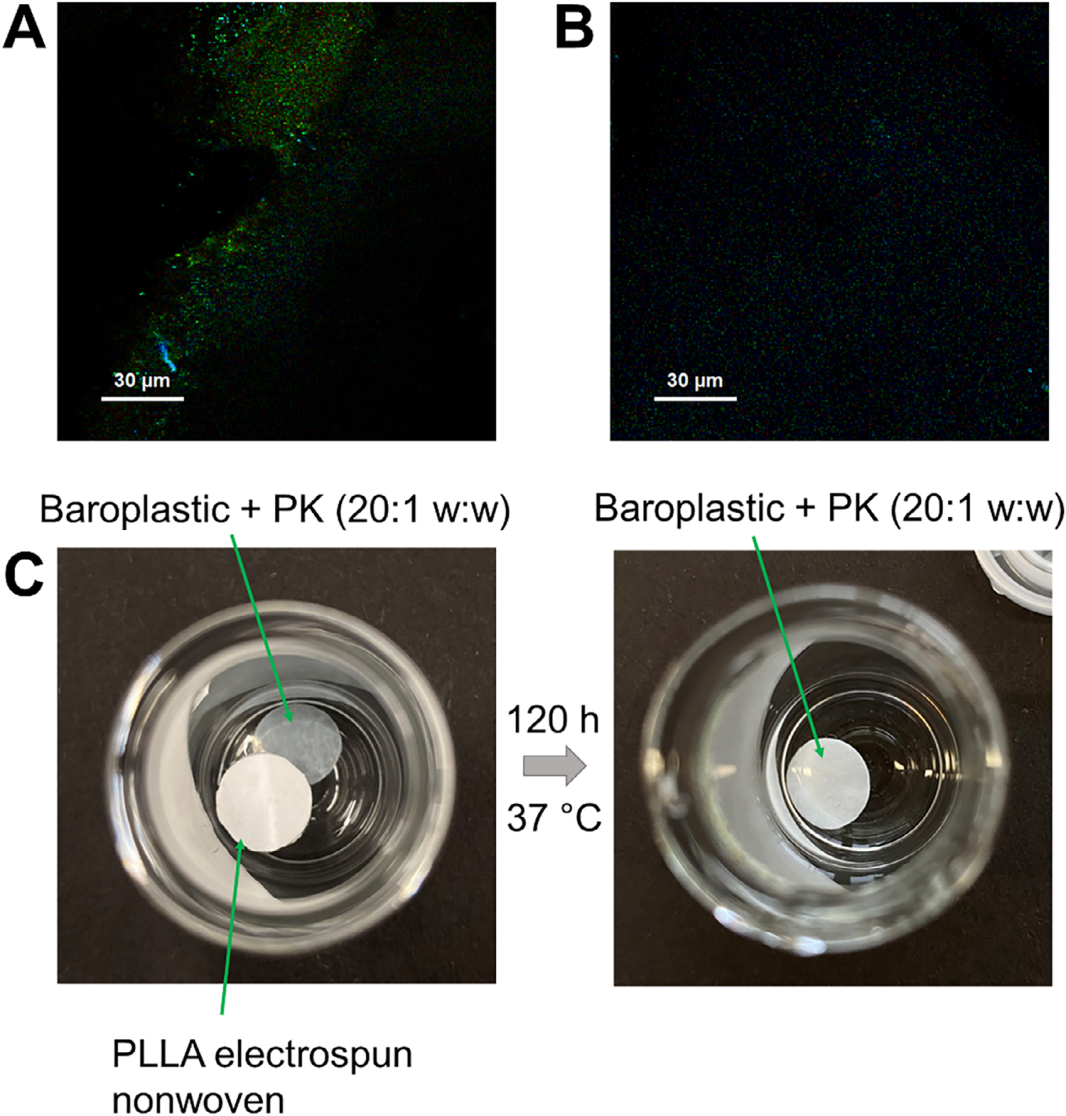
Protein encapsulation. Top: fluorescence microscopic images of YPet immobilized in PLLA_118_‐*b*‐PEG_200_‐*b*‐PLLA_118_ (A) under baroplastic conditions (film pressed at 37°C under 10 MPa for 5 min), and (B) at 135°C under 10 MPa for 5 min (sample 21). The brightness of the color in the images represents the fluorescence intensity of YPet. (C) Photographic demonstration of the PK leaching test from the cold‐pressed composite of sample 21 (37°C/10 MPa/5 min) with a PLLA electrospun nonwoven. The PLLA electrospun nonwoven was fully degraded after 5 days of exposure to the PK leachate.

To challenge the potential application of the compostable baroplastic PLLA‐*b*‐PEG‐*b*‐PLLA as carriers for temperature‐sensitive additives, we encapsulated the yellow‐fluorescent YPet protein and proteinase K (PK) in the PLLA_118_‐*b*‐PEG_200_‐*b*‐PLLA_118_ triblock copolymer (sample 21) and evaluated their functionality. The triblock copolymer was mixed with the temperature‐sensitive proteins and processed into films under baroplastic conditions (37°C/ 10 MPa/ 5 min). For comparison, films were also prepared by conventional high‐temperature processing (135°C/ 10 MPa/ 5 min). Fluorescence lifetime of the protein before and after processing can provide valuable insights. This parameter is sensitive to the local environment of the fluorophore and therefore to the conformation of the protein. If the native conformation of the protein is preserved within the polymer film, it is highly likely that neither the fluorescence quantum yield (i.e. the emitted intensity) nor the fluorescence lifetime will change significantly. In contrast, for a denatured protein it is expected that these parameters will be altered. The fluorescence of YPet encapsulated in the triblock copolymer and processed under baroplastic conditions was retained (Figure 5A; Figure S17). By contrast, the sample processed at 135°C for 5 min showed a near‐complete loss of yellow fluorescence indicating degradation of the majority of the protein (Figure 5B). A similar maintenance of activity was observed for PK at baroplastic processing conditions. To investigate this further, a composite film was prepared by mixing powdered PLLA_118_‐*b*‐PEG_200_‐*b*‐PLLA_118_ (sample 21) and PK in a mass ratio of 19:1 and processing the mixture at 37°C, 10 MPa for 5 min. In the literature, encapsulation of PK in an ambiently processable diblock copolymer of polyphosphoester and PLLA was previously reported, where functionality after processing was demonstrated, but enzymatic activity was not discussed [34]. To assay PK activity in this work, the composite film was incubated in a Tris buffer solution (pH 8.6), and the buffer was analyzed for enzymatic activity at defined time intervals. To the best of our knowledge, there is currently no established method to directly assess PK activity within a solid polymer matrix.; Therefore, enzymatic activity was evaluated indirectly via the enzyme released into the buffer solution. As an active PK hydrolysis chromogenic substrate (*N*‐Suc‐Phe‐Ala‐Ala‐Phe‐pNA) [35], the buffer solution with active enzyme should also hydrolyze the chromogenic substrate. After 21 days, the activity of the leached PK amounted to 40% of the activity of the enzyme initially used for the film preparation (Figures S18 and S19). In contrast, PK dissolved in Tris buffer without encapsulation exhibited a complete loss of activity after 15 days. Not all of the enzyme could be leached out, as a fraction remained encapsulated within the bulk polymer matrix, which may require longer times for complete release. In order to get a complete picture, as an additional experiment, we incubated an electrospun PLLA nonwoven in the presence of PK‐infused baroplastically processed PLLA_118_‐*b*‐PEG_200_‐*b*‐PLLA_118_ film (Figure 5C) and followed the enzymatic degradation of the PLLA nonwoven. The release of enzyme from the PLLA_118_‐*b*‐PEG_200_‐*b*‐PLLA_118_ was expected to degrade the PLLA nonwoven as it is known from the literature that PLLA electrospun nonwoven degrades faster than PLLA bulk film [36]. The PLLA nonwoven was completely degraded within 5 days, providing clear evidence that PK retained its enzymatic activity after encapsulation within the baroplastic matrix, thereby enabling effective enzymatic degradation on top of its inherent compostability.

## 3 Conclusions

The PLLA‐*b*‐PEG‐*b*‐PLLA triblock copolymers, which are widely used in biomedical applications, result in sustainable polymers with a unique combination of processability under baroplastic conditions with good thermomechanical stability, industrial compostability, environmental compatibility, and physical as well as chemical recyclability. The compostability of the PLLA‐*b*‐PEG‐*b*‐PLLA triblock copolymers has been demonstrated quantitatively by respirometry based on CO_2_ formation, which is distinct from their well‐known physiological degradation. The ability of PLLA‐*b*‐PEG‐*b*‐PLLA to be recycled under mild conditions minimizes material defects, which are otherwise common problems in the melt recycling of polymers. Owing to this advantageous property profile, PLLA‐*b*‐PEG‐*b*‐PLLA is of particular interest for various applications as a pristine material or as a carrier for heat‐sensitive functional additives. These polymers can, for instance encapsulate proteins that retain activity after processing. The quantitative results of this study on environmentally degradable baroplastic polymers might open a new research direction. Moreover, the versatile synthetic routes available for these polymers enable large‐scale production, thereby supporting both the advancement of this emerging research field and their implementation in real‐world applications.

## 4 Experimental Section

### 4.1 Materials

Dichloromethane (DCM, technical grade), diethyl ether (technical grade), stannous octoate (Sn(Oct)_2_; Thermo Scientific, Germany), chloroform (VWR, Germany), native proteinase K (PK, lyophilized powder from Tritirachium album, 28.9 kDa; GeneOn, Germany; stored at −20°C), tris(hydroxymethyl)aminomethane (Tris, 1 m buffer solution, pH 9.0; abcr, Germany), *N*‐succinyl‐alanine‐alanine‐proline‐leucine‐p‐nitroanilide (*N*‐Suc‐Ala‐Ala‐Pro‐Leu‐pNA; Sigma–Aldrich, Germany), and aniline (≥99.5%; Carl Roth, Germany) were used as received. 1‐Decanol (synthesis grade; Merck, Germany) was distilled prior to use. Poly(ethylene glycol) methyl ether (mPEG 5000), PEG 8000, and PEG 20000 (Sigma–Aldrich, Germany), as well as *L*‐lactide and *D*,*L*‐lactide (PURASORB L and PURASORB DL; Corbion, The Netherlands), were dried under vacuum for 12 h prior to polymerization. Tris buffer was diluted with ultrapure water (Milli‐Q Reference A+) to a final concentration of 50 mm, and the pH was adjusted to 8.6 using hydrochloric acid (37%; VWR, Germany).

The *Daphnia magna* clone BL2.2 used in this study was originally collected from a small pond (Oud Meren, Leuven, Belgium) and has been maintained under continuous laboratory culture since 1997. The organisms were reared in M4 medium at 20 ± 0.5°C under a 16 h light / 8 h dark cycle and fed *Acutodesmus obliquus* ad libitum. In accordance with OECD guideline 202, the clone was routinely tested (at least twice a year) for acute toxicity using sodium chloride as a reference substance. The resulting EC_50_ (48 h) value of 4.925 µg mL^−^^1^ is consistent with values reported for this clone in related studies, confirming its suitability for acute toxicity testing.

The murine macrophage cell line J774A.1 (ACC170), the human adenocarcinoma epithelial cell line Caco‐2 (ACC169), and human macrophages derived from differentiation of the monocytic cell line THP‐1 (ACC16) were obtained from DSMZ (Braunschweig, Germany). Cells were cultured in T‐75 flasks (Corning, USA) at 37°C under a humidified atmosphere containing 5% CO_2_. J774A.1, Caco‐2, and THP‐1 cells were passaged three, two, and twice a week, respectively. J774A.1 cells were cultured in Dulbecco's Modified Eagle Medium (DMEM) supplemented with 10% heat‐inactivated fetal bovine serum (FBS). Caco‐2 cells were maintained in DMEM supplemented with 10% heat‐inactivated FBS, and THP‐1 cells were cultured in Roswell Park Memorial Institute medium (RPMI 1640) supplemented with 10% heat‐inactivated FBS. Prior to experiments, J774A.1 cells were detached by scraping, collected in culture medium, centrifuged (200 g, 2 min, 20°C), and resuspended in fresh medium. Caco‐2 cells were washed with Dulbecco's phosphate‐buffered saline (DPBS) and collected by trypsinization for 2 min at 37°C. THP‐1 cells were transferred into fresh culture medium prior to use.

### 4.2 Polymerization

Ring‐opening polymerization (ROP) was conducted in a flame‐dried and argon‐purged Schlenk tube. Different amounts of mPEG/PEG and lactide (Table S1) were dried under reduced pressure for 12 h, then Sn(Oct)_2_ was added into the reaction mixture, corresponding to a monomer‐to‐catalyst molar ratio of 17,500:1 and the reaction temperature was kept at 140°C for 6 h. After the reaction, the product was dissolved in DCM and precipitated in cold diethyl ether (cooled by an ice bath at 0°C). After filtration, the polymers were dried under vacuum at 37°C for 24 h.

### 4.3 Polymer Characterization

^1^H nuclear magnetic resonance (^1^H NMR) spectra were recorded on a Bruker Ultrashield 300 spectrometer using CDCl_3_ as solvent. The residual non‐deuterated solvent signal was used as an internal reference.

Thermal properties of the synthesized polymers were characterized by differential scanning calorimetry (DSC) using a NETZSCH DSC 204 F1 Phoenix. Measurements were performed under a nitrogen atmosphere (flow rate 20 mL min^−^^1^) at a heating rate of 10 K min^−^^1^.

Gel permeation chromatography (GPC) was conducted using a system equipped with a styrene–divinylbenzene (SDV) precolumn (5 µm, PSS Mainz), an SDV linear XL column (5 µm, PSS Mainz), and a refractive index detector (Agilent Technologies). Samples were dissolved in CHCl_3_ containing toluene (HPLC grade) as an internal standard at a concentration of 2 mg mL^−^^1^, filtered through a 0.22 µm polytetrafluoroethylene (PTFE) filter (BGB, China), and analyzed at room temperature with a flow rate of 0.5 mL min^−^^1^. Calibration was performed using narrowly distributed polystyrene standards (PSS calibration kit, PSS Standard Service). For PEG molecular weight determination, PEG standards in the range of 1,000 – 26,000 Da were used. The viscosity‐average molecular weight of PEG was additionally determined using a Ubbelohde viscometer based on PEG solutions with concentrations ranging from 6.6 to 10 mg mL^−^^1^, with each measurement performed in ten replicates.

X‐ray diffraction (XRD) measurements were carried out in Bragg–Brentano geometry using an Empyrean diffractometer (Malvern Panalytical BV) equipped with a pixel detector and Cu *K*_α_ radiation (*λ* = 1.54 Å). Small‐angle x‐ray scattering (SAXS) experiments were performed using a laboratory‐based Ganesha AIR system (SAXSLAB/Xenocs) equipped with a copper rotating anode (MicroMax 007HF, Rigaku; *λ* = 1.54 Å) and a Pilatus 300K detector (Dectris Ltd.). 2D scattering patterns were converted to 1D intensity profiles *I*(*q*) vs. *q*, where *q* = (4π/*λ*) sin(*θ*/2) and *θ* is the scattering angle. Data analysis was performed using the instrument software for XRD and SAXS utilities for SAXS.

Transmission electron microscopy (TEM) was performed at an acceleration voltage of 200 kV using a JEOL JEM‐2200FS microscope equipped with a field‐emission gun and an in‐column Ω energy filter. Zero‐loss energy‐filtered micrographs were recorded under cryogenic conditions (holder temperature −176°C) using a bottom‐mounted CMOS 4K camera (OneView, Gatan). Image processing was carried out using Digital Micrograph 3.5 (Gatan) and the Contrast Limited Adaptive Histogram Equalization (CLAHE) plugin in ImageJ (block size 150, histogram bins 256, maximum slope 1.5). For TEM sample preparation, bulk specimens were plunge‐frozen in liquid nitrogen and sectioned into ultrathin slices at −100°C using a Leica UC7 ultramicrotome equipped with an EM FC7 cryo‐chamber (Leica, Germany). Sections were transferred onto lacey carbon‐coated copper grids (S166 Plano, Germany). To prevent water contamination during warming, the grids were transferred under liquid nitrogen into a high‐vacuum chamber. For selective staining, samples were exposed to RuO_4_ vapor for 10 min, generated in situ from RuCl_3_ hydrate and NaOCl. The stained grids were plunge‐frozen in liquid nitrogen and transferred under cryogenic conditions to the TEM for analysis.

### 4.4 Protein Expression and Purification

The light‐regulated pREDawn‐YPet system encoding YPet with an N‐terminal 6×His tag was constructed as described previously [37]. The pREDawn‐YPet plasmid was chemically transformed into *Escherichia coli* CmpX13. *E. coli* CmpX13 cells harboring pREDawn‐YPet were cultivated in lysogeny broth (LB) medium supplemented with kanamycin (50 µg mL^−^^1^) at 37°C and 225 rpm under dark conditions. When the optical density at 600 nm reached 0.6, cells were illuminated with 660 nm light at an intensity of 100 µW cm^−^^2^ for 18 h while maintaining the cultivation temperature at 37°C.

After incubation, cells were harvested by centrifugation and lysed by sonication. The lysate was clarified by centrifugation and the supernatant was purified by Co^2^^+^ immobilized metal ion affinity chromatography (IMAC). His‐tagged YPet was eluted using an imidazole gradient ranging from 20 to 500 mm. Elution fractions were analyzed by denaturing polyacrylamide gel electrophoresis (PAGE), and fractions with sufficient purity and yield were pooled and dialyzed overnight at 4°C against Tris buffer (50 mm Tris/HCl, 20 mm NaCl, 5 mm β‐mercaptoethanol, pH 8.0). Following dialysis, the purified YPet protein was lyophilized to obtain a dry powder.

### 4.5 Polymer Processing

Polymer films were prepared using a P/O Weber‐H HDP300 hydraulic press. Samples were pressed at a pressure of 10 MPa at either 37°C or 135°C. The materials were placed between two sheets of reusable glass fiber–reinforced PTFE paper (High‐Tech‐Flon, Germany). Films pressed at 135°C were cooled to room temperature using circulating cooling water at 22°C. For processing block copolymers with YPet, a polymer‐to‐protein weight ratio of 99:1 was employed. YPet was coated onto PLLA_118_‐*b*‐PEG_200_‐*b*‐PLLA_118_ (sample 21) powder using an airbrush, and the coated samples were pressed at 37°C or 135°C for comparison. For PK processing, 15 mg of PK powder was mixed with 300 mg of sample 21, corresponding to a polymer‐to‐protein weight ratio of 20:1. Subsequently, 6 mL of ultrapure water (Milli‐Q, Merck) was added, and the mixture was vortexed at 60 rpm for 10 min. The water was removed by freeze–drying, and the resulting powder was pressed at 37°C under a pressure of 10 MPa for 5 min.

### 4.6 Fluorescence Microscopy

Fluorescence lifetime experiments were performed using a commercial MicroTime 200 optical microscope (PicoQuant). The setup was equipped with a pulsed laser diode operated at a repetition rate of 20 MHz, providing an excitation wavelength of 482 nm (LDH‐D‐C‐485, PicoQuant). The spectral output of the laser was cleaned using a bandpass filter (ET480/20m, AHF/Croma). Laser radiation was coupled into the main optical unit via a single‐mode optical fiber and reflected by a dichroic mirror (ZT405/485rpc, AHF/Croma) into an inverted confocal microscope, where it was focused onto the sample using an objective (MPLFLN100, NA = 0.9, Olympus). Fluorescence lifetime images were acquired by raster scanning the focal spot over an area of 150 µm × 150 µm using a galvo scanner (FLIMbee unit, PicoQuant). Emission from the sample was directed back through the dichroic mirror and passed through a long‐pass filter (488 LP Edge Basic, Semrock). The fluorescence signal was detected using a single‐photon counting avalanche photodiode (SPCM‐AQRH‐14‐TR, Excelitas). Time‐correlated single‐photon counting (TCSPC) was performed using a TimeHarp 260 PICO Dual module (PicoQuant) with a temporal resolution of 250 ps, operating in time‐tagged time‐resolved (TTTR) mode. The recorded fluorescence decay transients were deconvoluted with the instrument response function and fitted using the commercial software SymPhoTime 64 (PicoQuant).

### 4.7 Tensile Test

Pressed films were cut into tensile test specimens using a hydraulic sample punch in accordance with DIN 53 504 S3A (Coesfeld Materialtest, Germany). The thickness of each specimen was measured using a digital micrometer (Series, 0–25 mm; Mitutoyo, Neuss, Germany), and the average value was calculated from three measurements taken at different positions within the gauge length. Tensile tests were performed using a universal testing machine (BT1‐FR0.5TN.D14, Zwick/Roell, Germany). A crosshead speed of 0.2 mm min^−^^1^ was applied in the Young's modulus region, followed by a crosshead speed of 1 mm min^−^^1^ for the remainder of the test.

### 4.8 Wastewater/Compost Biodegradation Monitored by Micro‐Oxymax

Wastewater biodegradation tests were performed using a Micro‐Oxymax respirometer (Columbus Instruments International, USA). Samples were tested in triplicate over a period of 42 days in accordance with DIN ISO 14851:2019. Activated sludge water (post‐nitrification) was collected from the Bayreuth wastewater treatment plant and used as the inoculum. Approximately 95 mg of sample was added to 100 mL of test medium consisting of 95 mL of standard medium and 5 mL of activated sludge supernatant. Blank experiments contained the same concentration of activated sludge without test material, and aniline was used as a positive reference. The Micro‐Oxymax system, equipped with calibrated CO_2_ and O_2_ sensors, an infrared (IR) CO_2_ detector, a sample pump, air supply units, gas‐drying columns (≥98% CaSO_4_, <2% CoCl_2_), 0.2 µm particle filters, and condensers, was used to monitor CO_2_ evolution at defined intervals. All samples were incubated at 25°C in a closed‐loop configuration.

Aerobic composting biodegradation was evaluated under ASTM D5338 conditions using the same Micro‐Oxymax respirometer. Compost aged for 12 weeks was collected from the “Am Buchstein” facility (Bayreuth, Germany), sieved to <1 cm, and subsequently characterized in terms of pH, moisture content, ash content, and total carbon and nitrogen. Films of sample 20 prepared under baroplastic processing conditions (37°C, 10 MPa, 1 h) were cut into 15 × 15 mm^2^ specimens with a thickness of 500 µm, and 2 g of film was mixed with 200 g of compost adjusted to 50 wt.% water content. Poly(*L*‐lactide) (PLLA) films of identical dimensions were used as reference materials. Blank compost samples served as negative controls and sodium benzoate was employed as the positive reference. Experiments were conducted in triplicate at 58°C for 60 days, and CO_2_ accumulation was quantified using the IR sensor.

Total organic carbon (TOC) analysis was performed to calculate the theoretical CO_2_ evolution (ThCO_2_) for the determination of biodegradation percentages. Measurements were carried out using a multi N/C 3100 analyzer (Analytik Jena) equipped with an HT 1300 solid module and calibrated with potassium phthalate. For each analysis, 10 – 20 mg of sample was weighed into an Al_2_O_3_ crucible and combusted at 1,200°C in an oxygen stream. The resulting CO_2_ was transported to the detector via an integrated pump and quantified using a non‐dispersive infrared (NDIR) detector. All TOC measurements were performed in duplicate. The biodegradation percentage was calculated according to Equation (1):

(1) $$Biodegradation\%=\frac{CO_{2},\;sample-CO_{2},\;blank}{Th_{CO_{2}}}\;\times 100$$

where *CO_2,sample_* is the amount of CO_2_ evolved from the test material flask (mg), and *CO_2,blank_* is the amount of CO_2_ evolved from the blank flask from the start to the end of the test (mg). *Th_CO2_* is the theoretical amount of CO_2_ evolved from the test material (mg), calculated as Equation (2):

(2) $$Th_{CO_{2}}\;\left[mg\right]=m{,}_{sample}\;\times TOC\;\times \frac{M_{CO_{2}}}{M_{C}}$$

Where, *m,_sample_* is the mass of the sample, $M_{{\mathrm{CO}}_{2}}$ and *M*_C_ are the molar masses of CO_2_ and carbon, respectively, and *TOC* is the carbon content of the specimen determined by total organic carbon analysis.

### 4.9 *Daphnia* Acute Toxicity Test

Sample 21 was first dissolved in tetrahydrofuran (THF) with a concentration of 120 mg/mL. 1 mL of the baroplastic/THF solution was then dispersed in 2 mL M4 medium by a solvent replacement method (adding the baroplastic/THF solution dropwise into M4 medium under constant stirring at 1,000 rpm) to get particles with a size of 288.63 ± 1.63 nm for *Daphnia* acute toxicity test with a final concentration of 60 mg/mL. The particle size was determined by dynamic light scattering (DLS). A Zetasizer Nano S (Malvern Panalytical, UK) device with a red He─Ne laser (*λ* = 632.8 nm) and a detector positioned at θ = 173 ° to the incident radiation was used to perform DLS. In order to prevent solvent evaporation, the sample suspension was measured in a quartz glass cuvette (10.0 mm path length) that was covered with a plastic lid and kept at 25°C. The Malvern Zetasizer software (version 7.13) was utilized for conducting data analyses. Particle size is provided as hydrodynamic diameter, which is calculated from the average of three replicate observations.

Third brood neonates (≤ 24 h) were used for the experiment. These age‐synchronized animals were randomly distributed into groups of five individuals, each placed in 10 mL of M4 medium within individual sample wells of a 6‐well plate. A total of 120 animals were subjected to exposure to five different concentrations of the sample 21 (31.25, 62.5, 125, 250, and 500 µg/mL + control (0 µg/mL)), each with four independent replicates. Following the OECD Guideline 202, no supplementary food was provided, and no medium exchanges were performed during the exposure period. The experiment was extended to a duration of 96 h as recommended by Baumann et al.^31^, with the animals being visually assessed for immobilization at 24, 48, and 96 h.

### 4.10 Alamar Blue Assay

Sample 21 was dispersed in ultrapure water (Milli‐Q Reference A+) by a solvent replacement method as described above to get particles for the Alamar blue viability cell test using a final concentration of 60 mg/mL.

Three cell lines were used to test for the potential cytotoxicity of the PLLA‐*b*‐PEG‐*b*‐PLLA triblock copolymer: the murine macrophage cell line J774A.1 (ACC170), the human adenocarcinoma epithelial cell line Caco2 (ACC169), and human macrophages derived from the differentiation of monocytic cells THP‐1 (ACC16). Each cell line was counted using a hemocytometer (Neubauer improved, Brand, Wertheim, Germany). For each treatment, 6 sample wells of a 96‐well plate (CellStar, Greiner Bio‐One, Frickenhausen, Germany) for each cell line in 100 µL of the corresponding cell culture medium were tested. Since the cell lines have different proliferation rates, the J774A.1 were seeded as 50,000 cells/mL‐forming units, and for Caco2 and THP‐1, 100,000 cells/mL‐forming units were added and allowed to adhere under standard culture conditions (37°C, 5% CO_2_, humidified) overnight. The THP‐1 were differentiated into macrophages using 100 mm 12‐O‐tetradecanoylphorbol‐13‐acetate (TPA).

The cells were treated with increasing concentrations of sample 21 suspension to 0 (control), 0.1, 1, 10, 100, 500, and 1,000 µg/mL and with a 0.2% Triton X solution as a positive control treatment. The cells were incubated for 24 h under cell culture conditions, and subsequently, 10 µL of the Alamar blue dye (Invitrogen, ThermoFisher Scientific) was added and further incubated for 5 h. The well plates were measured at *λ =* 530 – 560 nm excitation and *λ =* 590 nm emission using a plate reader (Varioskan LUX, ThermoFisher Scientific). The experiments were performed three times and the data is shown as blank‐subtracted mean values of the three individual experiments in percentage in relation to control viability (**≙** 100%).

The statistical analysis was performed in R Studio [32] using the packages “rstatix”, “stats”, and “rcompanion”. The data was tested for normal distribution and heteroscedacity and subsequently a Kruskal‐Wallis test with a Games‐Howell post hoc test was performed.

### 4.11 Recycling

Physical recycling of PLLA_93_‐*b*‐PEG_200_‐*b*‐PLLA_93_ (sample 20) was performed using a cryomill (ZM300, Retsch). The sample was first pressed under baroplastic conditions (10 MPa, 37°C, 14 h), cryomilled in liquid nitrogen for 30 s, and the resulting powder was repressed under the same conditions.

Chemical recycling was conducted using a film of PLLA_93_‐*b*‐PEG_200_‐*b*‐PLLA_93_ (sample 20). The film was pressed at 80°C under 10 MPa for 3 min, then subjected to hydrolytic degradation by immersion in 0.5 m aqueous sodium hydroxide. The mixture was incubated at 37°C in a shaking incubator at 60 rpm for 48 h. After incubation, water was removed by freeze–drying, and the solid residue was extracted with either chloroform or diethyl ether.

### 4.12 Proteinase K Activity Test

The concentration of proteinase K was determined by absorption spectroscopy using an Agilent 8435 diode‐array spectrophotometer. The activity assay was performed in a 96‐well clear microtiter plate in a multi‐mode microplate reader (CLARIOstar, BMG Labtech) using *N*‐Suc‐Ala‐Ala‐Pro‐Leu‐pNA as substrate. The activity measurement was done at 25°C by following the absorbance change at 410 nm over time. The specific activity was calculated using Equation (3):

(3) Specificactivity=ΔA/(ε·h·Δt·C)

where *ΔA* represents the difference of absorbance at *λ =* 410 nm relative to time zero, *ε* is the molar extinction coefficient of *p*‐nitroaniline at a wavelength of *λ =* 410 nm (*ε* = 8,800 m ^−1^ cm^−1^), *h* is the thickness of the solution, *Δt* is the monitoring time, and *C* is the molar concentration of PK in the solution.

PK encapsulation was performed as described above in the ‘Polymer Processing’ section. A piece of the pressed film with a weight of 30 mg was immersed in 2 mL Tris buffer and incubated at 37°C in a water bath. After 504 h, the supernatant was taken out for the proteinase K leaching test. The calibration curve of the proteinase K was done with proteinase K/Tris buffer solution at different concentrations. The concentration of the calibration samples was determined by UV absorbance at *λ =* 280 nm. The calibration curve was plotted with proteinase K activity vs. proteinase K concentration, fitted by OriginLab 8 software. The specific activity was normalized by proteinase K concentration determined by absorption spectroscopy. The amount of proteinase K leached out in the supernatant was calculated using the amount of proteinase K encapsulated in sample 21 as 100%. PLLA was electrospun following a previously described method [20].

## Author Contributions

A.G., A.M., and S.A. conceived and designed the study. C.X. performed the synthesis, polymer characterization, polymer processing, enzymatic degradation, enzymatic assay, polymer recycling, and collected the data. C.Y. synthesized fluorescent protein (YPet). E.F. did the compost degradation, analyzed, and interpreted the data. A.FRM.R., J.B., C.L, and H.K. did a cell/daphnia toxicity test, analyzed, and interpreted the data. S.R. did SAXS, XRD, analyzed, and interpreted the data. U.M. did TEM, analyzed, and interpreted the data. C.D. did DLS, analyzed, and interpreted the data. H.S. analyzed and interpreted the data.

## Conflicts of Interest

The authors declare no conflicts of interest.

## Supporting information




**Supporting File**: smll73189‐sup‐0001‐SuppMat.docx.

## Data Availability

The data that support the findings of this study are available in the supplementary material of this article.
