# Supplementary material for: Compostable and Recyclable Baroplastic Triblock Copolymers Enable Low‐Energy Polymer Processing
Source: Small. 2026 Mar 30;22(37):e14939. doi: 10.1002/smll.202514939 (PMC13325701; doi:10.1002/smll.202514939)
Supplement: Supplementary file 1 — Supporting File: smll73189‐sup‐0001‐SuppMat.docx. [file SMLL-22-e14939-s001.docx]

Supporting information

**Compostable and Recyclable Baroplastic Triblock Copolymers Enable Low-Energy Polymer Processing**

**Authors:** Chengzhang Xu^1^, Chengwei Yi^2^, Emilia Fulajtar^1^, Anja FRM Ramsperger^3,5^, Julian Brehm^3^, Christian Laforsch^3^, Holger Schmalz^1^, Sabine Rosenfeldt^4^, Ulrich Mansfeld^4^, Holger Kress^5^, Andreas Möglich^2^*, Andreas Greiner^1^*, Seema Agarwal^1^*

**Affiliations:**

^1^Macromolecular Chemistry and Bavarian Polymer Institute, University of Bayreuth; Bayreuth 95440, Germany.

^2^Department of Biochemistry, University of Bayreuth; Bayreuth 95440, Germany.

^3^Animal Ecology I, University of Bayreuth; 95440 Bayreuth, Germany.

^4^Physical Chemistry 1 and Bavarian Polymer Institute, University of Bayreuth; 95440 Bayreuth, Germany.

^5^Biological Physics, University of Bayreuth; 95440 Bayreuth, Germany.

*Corresponding authors. Email: andreas.moeglich.uni-bayreuth.de, greiner@uni-bayreuth.de, agarwal@uni-bayreuth.de

**This file includes:**

Supplementary Text

Figs. S1 to S19

Tables S1 to S2

Supplementary Text

The molecular characteristics of PLLA, PDLLA, PEG, mPEG, and block copolymers are presented in Table S1.

**Table S1.** Thermal properties of the synthesized PLA-*b*-PEG block copolymers

| Sample | Polymer | *T_m_* (PEG)/  °C | *T_g_* (PLA)/  °C | *T_m_* (PLA)/  °C | Baroplastic^a)^ |
| --- | --- | --- | --- | --- | --- |
| 1 | mPEG_120_-*b*-PDLLA_49_ | 56 | - | - | - |
| 2 | mPEG_120_-*b*-PDLLA_142_ | 54 | - | - | - |
| 3 | mPEG_120_-*b*-PDLLA_188_ | 53 | - | - | - |
| 4 | mPEG_120_-*b*-PDLLA_308_ | 53 | - | - | - |
| 5 | mPEG_120_-*b*-PDLLA_406_ | 54 | - | - | - |
| 6 | mPEG_120_-*b*-PLLA_157_ | 56 | - | 163 | - |
| 7 | mPEG_120_-*b*-PLLA_261_ | 56 | - | 170 | - |
| 8 | PDLLA_15_-*b*-PEG_200_-*b*-PDLLA_15_ | 55 | - | - | - |
| 9 | PDLLA_54_-*b*-PEG_200_-*b*-PDLLA_54_ | 53 | - | - | - |
| 10 | PDLLA_110_-*b*-PEG_200_-*b*-PDLLA_110_ | 52 | - | - | - |
| 11 | PDLLA_129_-*b*-PEG_200_-*b*-PDLLA_129_ | 51 | - | - | - |
| 12 | PDLLA_200_-*b*-PEG_200_-*b*-PDLLA_200_ | 51 | - | - | - |
| 13 | PDLLA_259_-*b*-PEG_200_-*b*-PDLLA_259_ | 50 | - | - | - |
| 14 | PDLLA_319_-*b*-PEG_200_-*b*-PDLLA_319_ | 52 | - | - | - |
| 15 | PLLA_31_-*b*-PEG_200_-*b*-PLLA_31_ | 56 | - | - | - |
| 16 | PLLA_64_-*b*-PEG_200_-*b*-PLLA_64_ | 50 | - | 141 | - |
| 17 | PLLA_70_-*b*-PEG_200_-*b*-PLLA_70_ | 53 | - | 141 | - |
| 18 | PLLA_75_-*b*-PEG_200_-*b*-PLLA_75_ | 53 | - | - | + |
| 19 | PLLA_78_-*b*-PEG_200_-*b*-PLLA_78_ | - | - | 141 | + |
| 20 | PLLA_93_-*b*-PEG_200_-*b*-PLLA_93_ | 47 | - | 154 | + |
| 21 | PLLA_118_-*b*-PEG_200_-*b*-PLLA_118_ | 47 | - | 157 | + |
|  |  |  |  |  |  |
| 22 | PLLA_35_-*b*-PEG_455_-*b*-PLLA_35_ | 61 | - | - | - |
| 23 | PDLLA | - | 54 | - | - |
| 24 | PLLA | - | 62 | 160 | - |

a) +: shows baroplastic property with good film formation


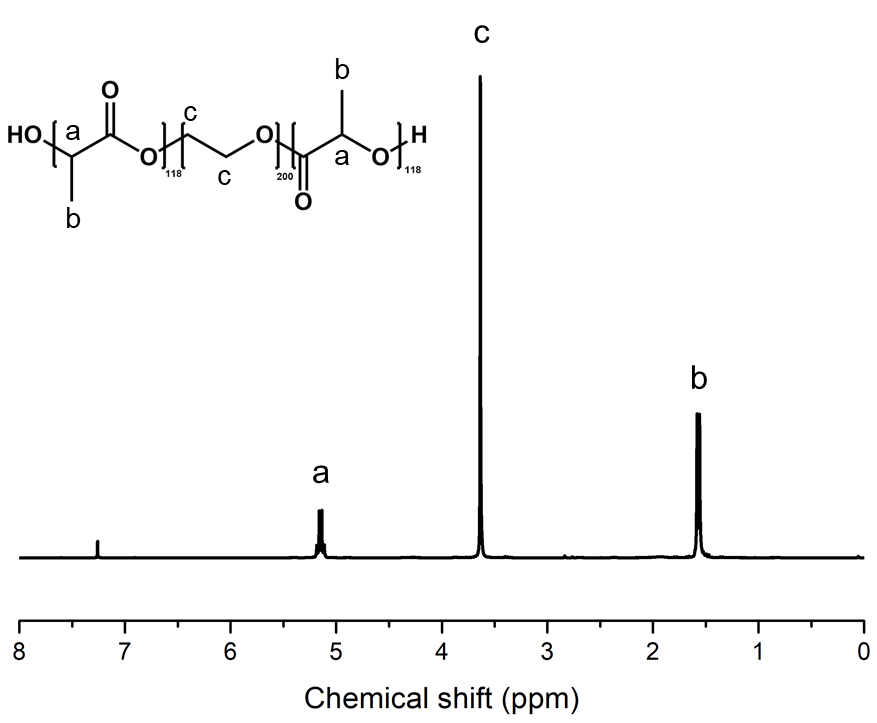


**FIGURE S1. ^1^H NMR spectrum for the triblock copolymer PLA_118_-*b*-PEG_200_-*b*-PLA_118_ (sample 21) as representative sample.**

The characteristic peaks for the PLLA (a, b: 1.6 ppm, 5.1 ppm) and the PEG (c: 3.6 ppm) blocks were clearly identified in ^1^H NMR as measured in CDCl_3_. The spectra of all diblock and triblock copolymers are shown in Fig. S2-S5. The number of lactic acid (LA) repeating units in the block copolymers was calculated based on Equations S1-S3:

$\text{I}_{\text{LA}}\text{ = }\text{I}_{\text{a}}$ (1)

$\text{I}_{\text{EG}}\text{ = }\text{I}_{\text{c}}\text{/4}$ (2)

$\text{n}_{\text{LA}}\text{ }\text{=}\text{ }\frac{\text{I}_{\text{LA}}}{\text{I}_{\text{EG}}}\text{ • }\text{n}_{\text{EG}}$ (3)

Where *n_LA_* is the number of lactic acid repeating units, *n_EG_* is the number of ethylene glycol repeating units calculated from the *M_n_* of the PEG precursors determined by GPC. *I_LA_* is the calculated integral of methine protons in the lactic acid repeating units, *I_EG_* is the integral of the methylene protons in the ethylene glycol repeating units normalized by 4 protons, *I_a_* is the integral of NMR peak at 5.1 ppm, *I_c_* is the integral of NMR peak at 3.6 ppm. The composition for all other block copolymers was calculated accordingly. In the used block copolymer nomenclature, the subscripts denote the average number of repeating units of the respective blocks. The experimental composition of most of the block copolymers compares well to the composition in feed for all educts except for mPEG_120_-*b*-PDLLA_49_, PDLLA_15_-*b*-PEG_200_-*b*-PDLLA_15_, and for PDLLA_54_-*b*-PEG_200_-*b*-PDLLA_54_ (Table S1). The reason for the deviation of the feed and experimental ratio could be explained by the extraction of unreacted mPEG homopolymer or block copolymers with short PLA blocks or sublimation of lactide during polymerization. The number average molecular weights ($\bar{M}_{\text{n}}$) of the block copolymer were calculated based on ^1^H NMR (Equation 4).

$\bar{M}_{\text{n}}\text{ = 72 g/mol • }\text{n}_{\text{LA}}\text{+}\bar{M}_{n}\text{(PEG)}$ (4)

Where 72 g/mol is the molar mass of LA repeating unit. *M_n_(PEG)* is the molecular weight of either PEG or mPEG used as macroinitiator. The molecular weight of the block copolymers calculated from NMR ranges from 8300 to 49900 g/mol.


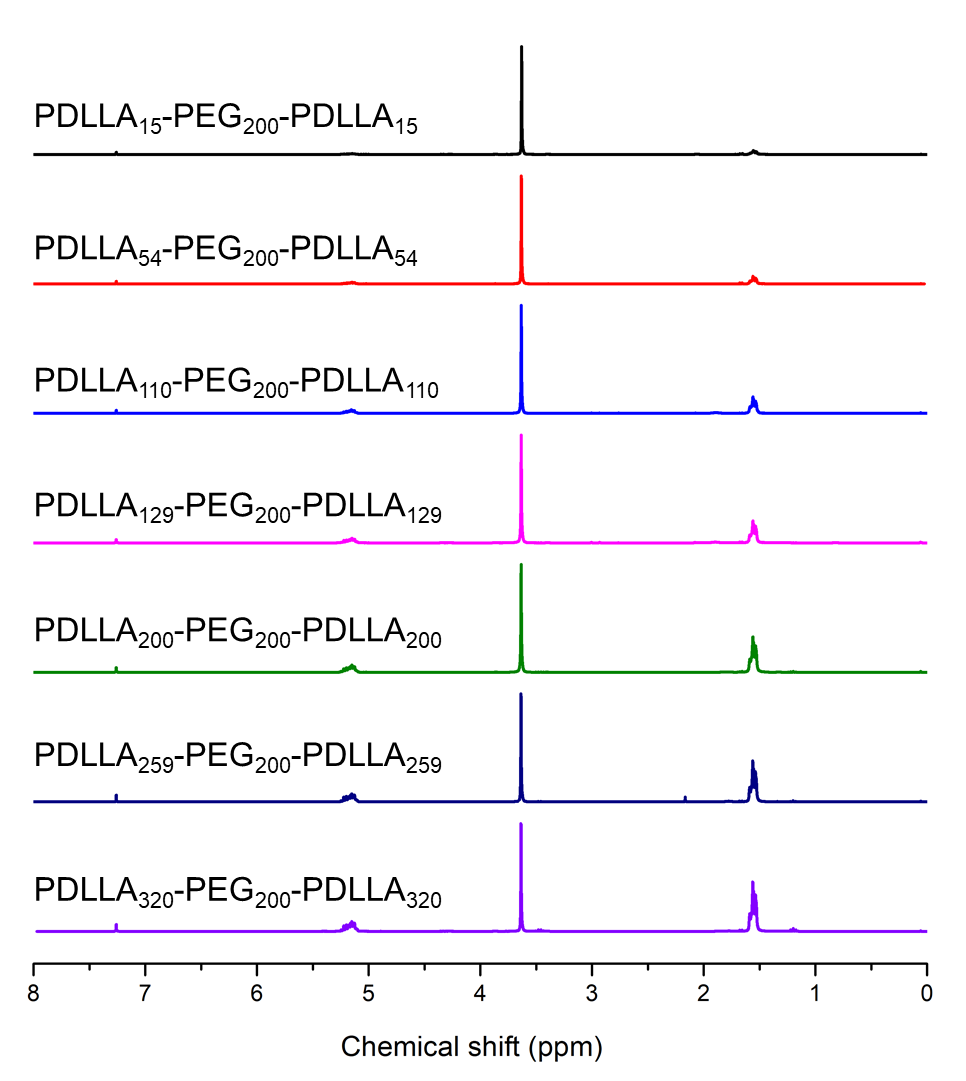


**FIGURE S2. ^1^H NMR spectra of PDLLA-*b*-PEG-*b*-PDLLA triblock copolymers.** The as-precipitated samples were dissolved in CDCl_3_, and the signal of the residual non-deuterated solvent was used as internal standard.


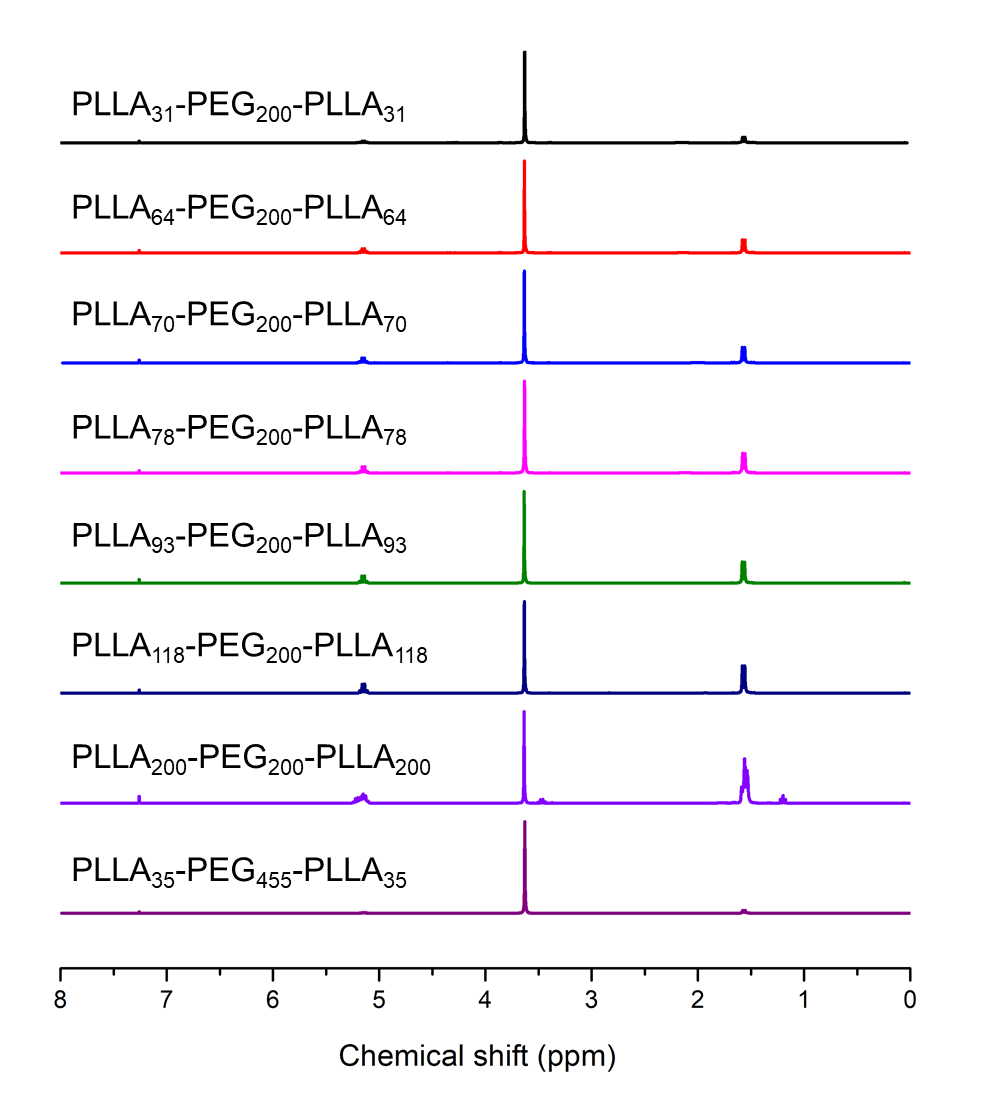


**FIGURE S3. ^1^H NMR spectra of PLLA-*b*-PEG-*b*-PLLA triblock copolymers.** The as-precipitated samples were dissolved in CDCl_3_, and the signal of residual non-deuterated solvent was used as internal standard.


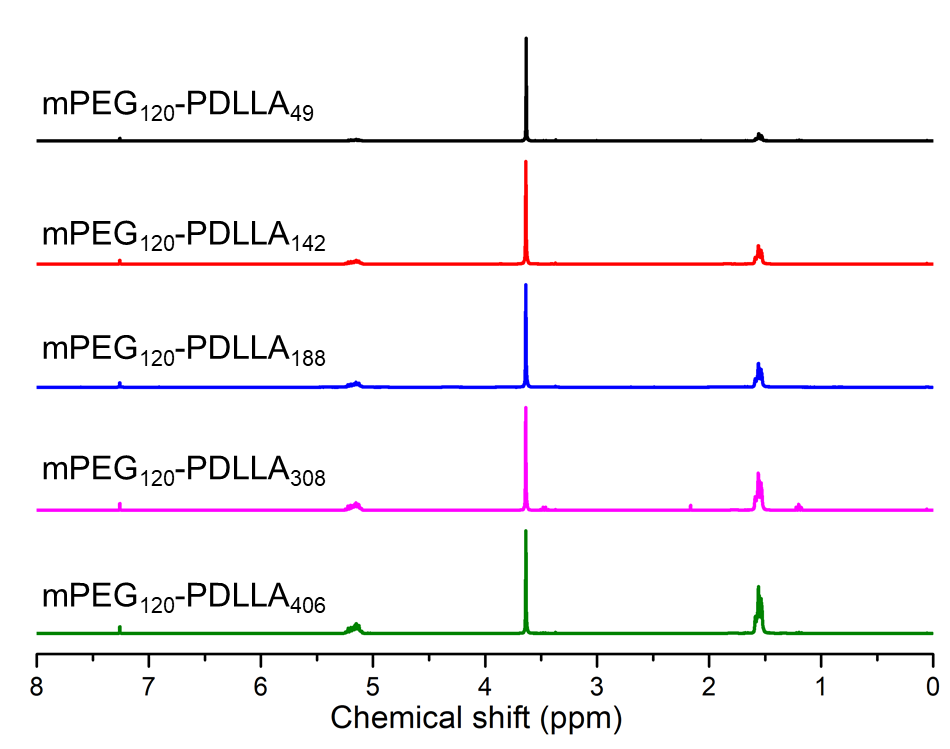


**FIGURE S4. ^1^H NMR spectra of mPEG-*b*-PDLLA diblock copolymers.** The as-precipitated samples were dissolved in CDCl_3_, and the signal of residual non-deuterated solvent was used as internal standard.


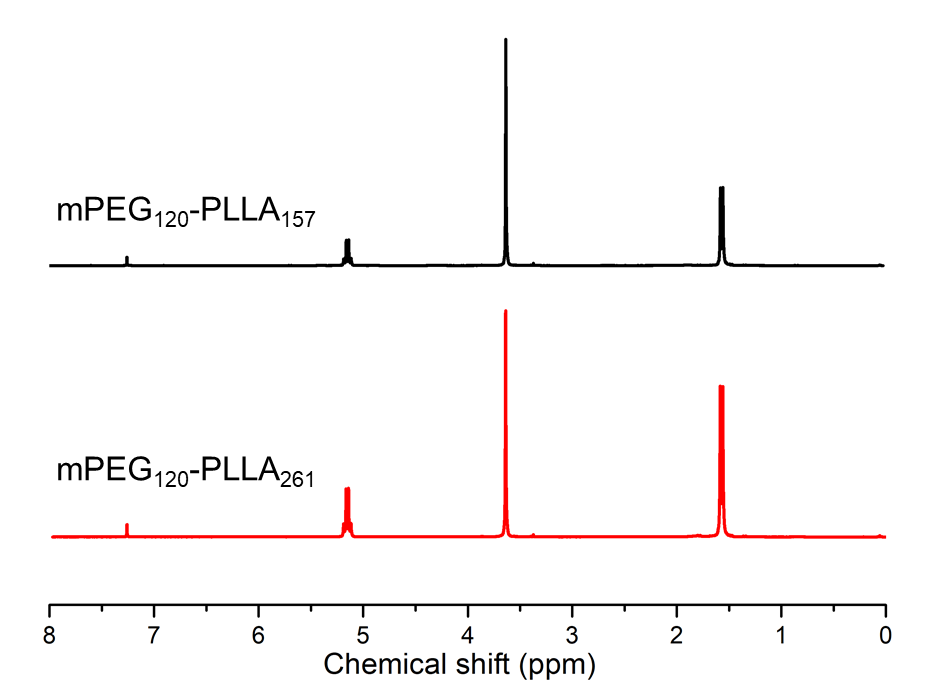


**FIGURE S5. ^1^H NMR spectra of mPEG-*b*-PLLA diblock copolymers.** The as-precipitated samples were dissolved in CDCl_3_, and the signal of residual non-deuterated solvent was used as internal standard.


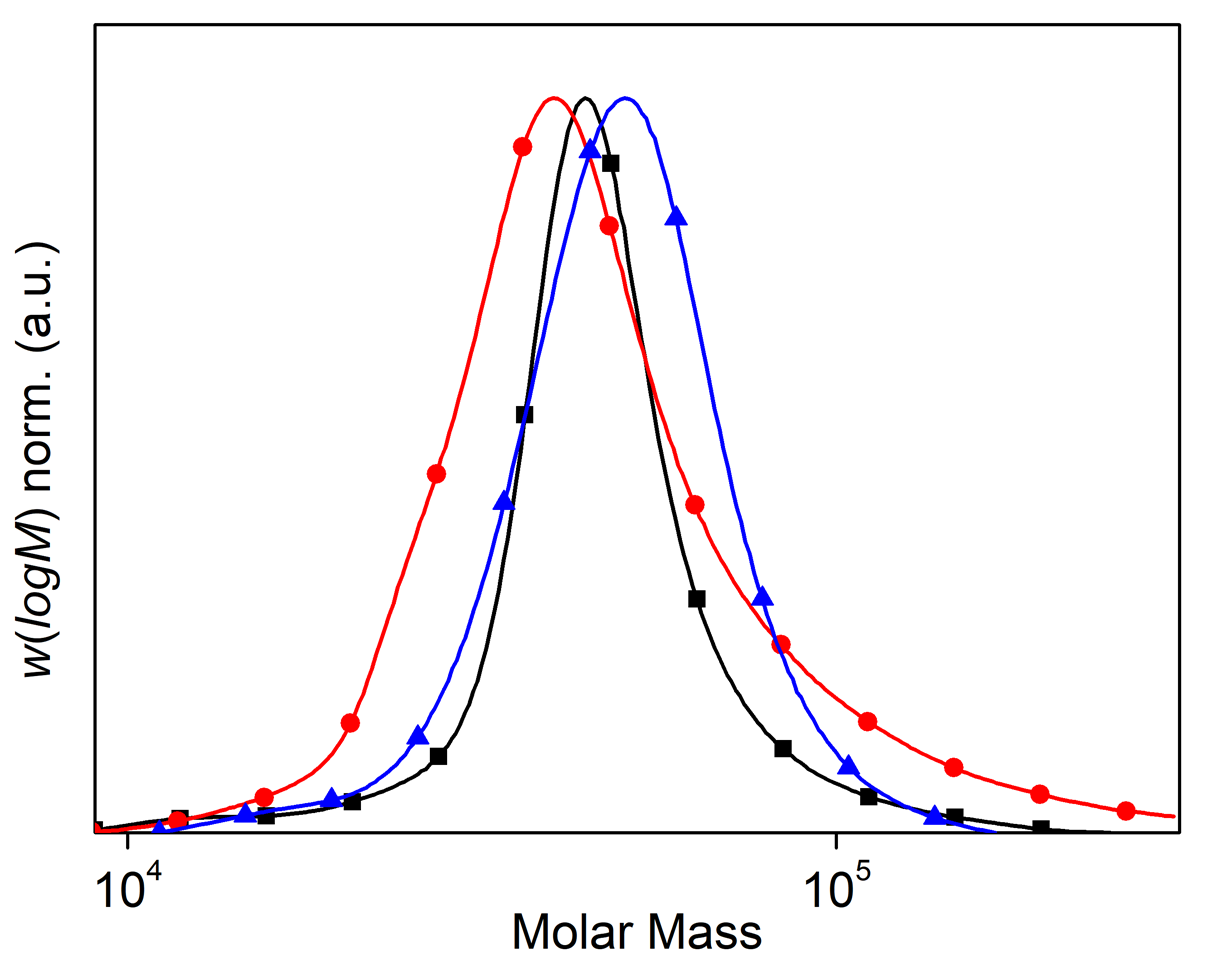


**FIGURE S6. Apparent molecular weight distribution of representative block copolymers.** Normalized molecular weight distributions of the mPEG_120_-*b*-PDLLA_142_ (sample 2, black square) diblock, as well as PDLLA_129_-*b*-PEG_200_-*b*-PDLLA_129_ (sample 11, red dot), and PLLA_118_-*b*-PEG_200_-*b*-PLLA_118_ (sample 21, blue triangle) triblock copolymers as determined by GPC.

The molecular weights of other samples were also determined by GPC and mentioned in Table1 (curves are not shown here).


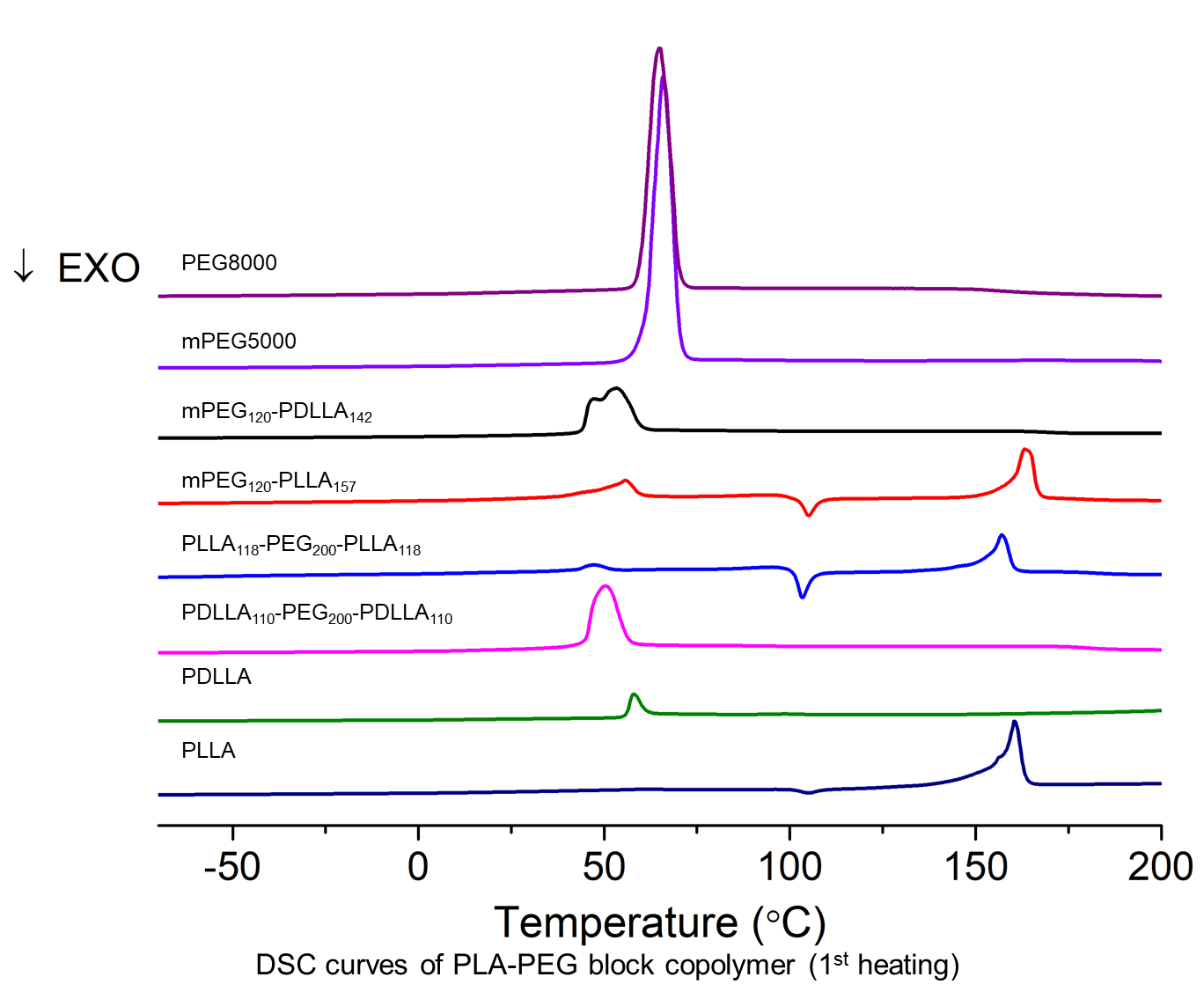


**FIGURE S7. First, heating curves of representative homopolymers and block copolymers.** DSC thermograms of PEG_200_ (purple), mPEG_120_ (violet), mPEG_120_-*b*-PDLLA_142_ (sample 2, black), mPEG_120_-*b*-PLLA_157_ (sample 6, red), PLLA_93_-*b*-PEG_200_-*b*-PLLA_93_ (sample 20, blue), PDLLA_110_-*b*-PEG_200_-*b*-PDLLA_110_ (sample 10, magenta), PDLLA (sample 24, green), and PLLA (sample 25, navy). Measurements were performed at a rate of 10 K/min under a nitrogen atmosphere with 20 mL per min flow.


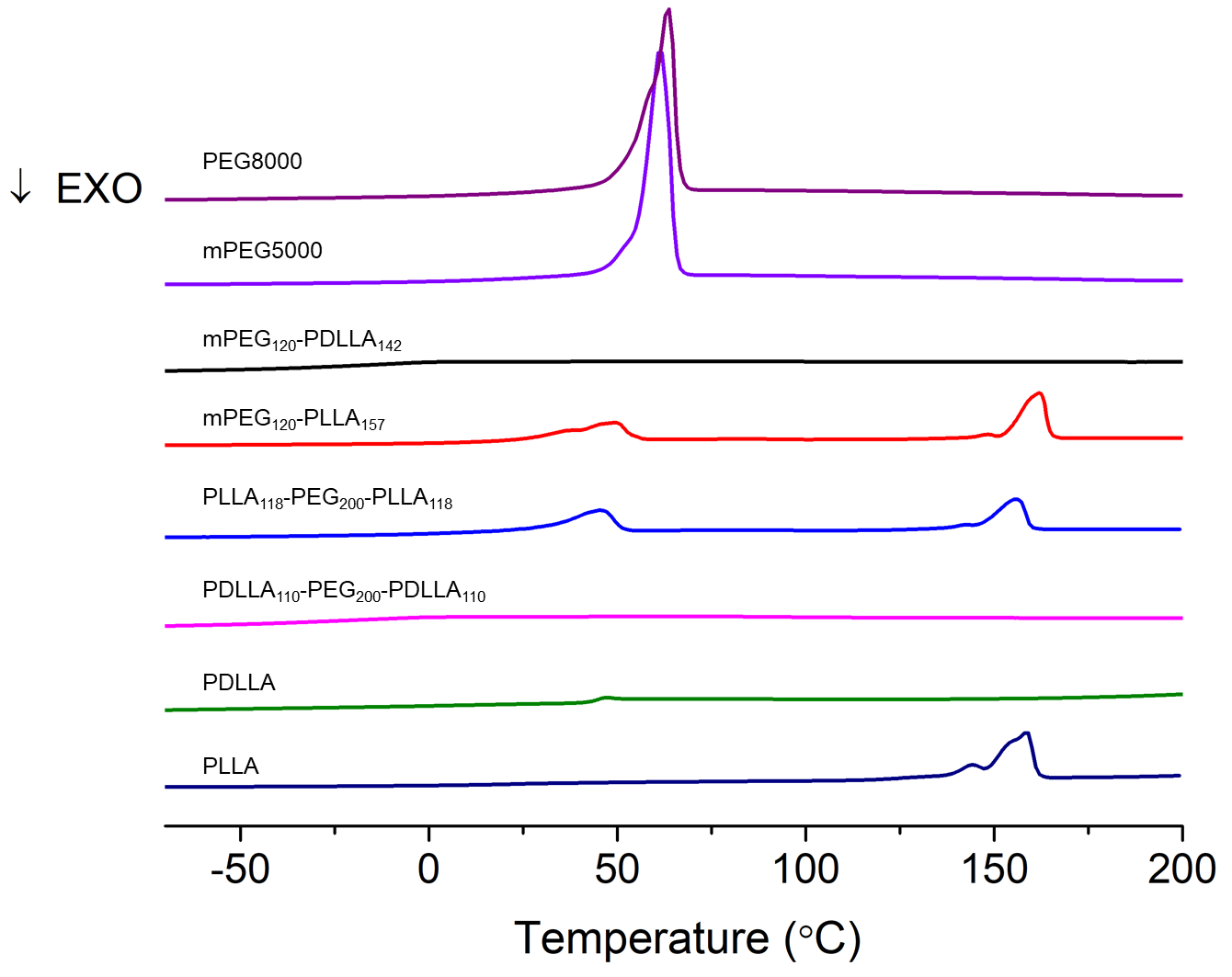


**FIGURE S8. Second, heating curves of the polymers.** DSC thermograms of PEG_200_ (purple), mPEG_120_ (violet), mPEG_120_-*b*-PDLLA_142_ (sample 2, black), mPEG_120_-*b*-PLLA_157_ (sample 6, red), PLLA_93_-*b*-PEG_200_-*b*-PLLA_93_ (sample 20, blue), PDLLA_110_-*b*-PEG_200_-*b*-PDLLA_110_ (sample 10, magenta), PDLLA (sample 24, green), and PLLA (sample 25, navy).


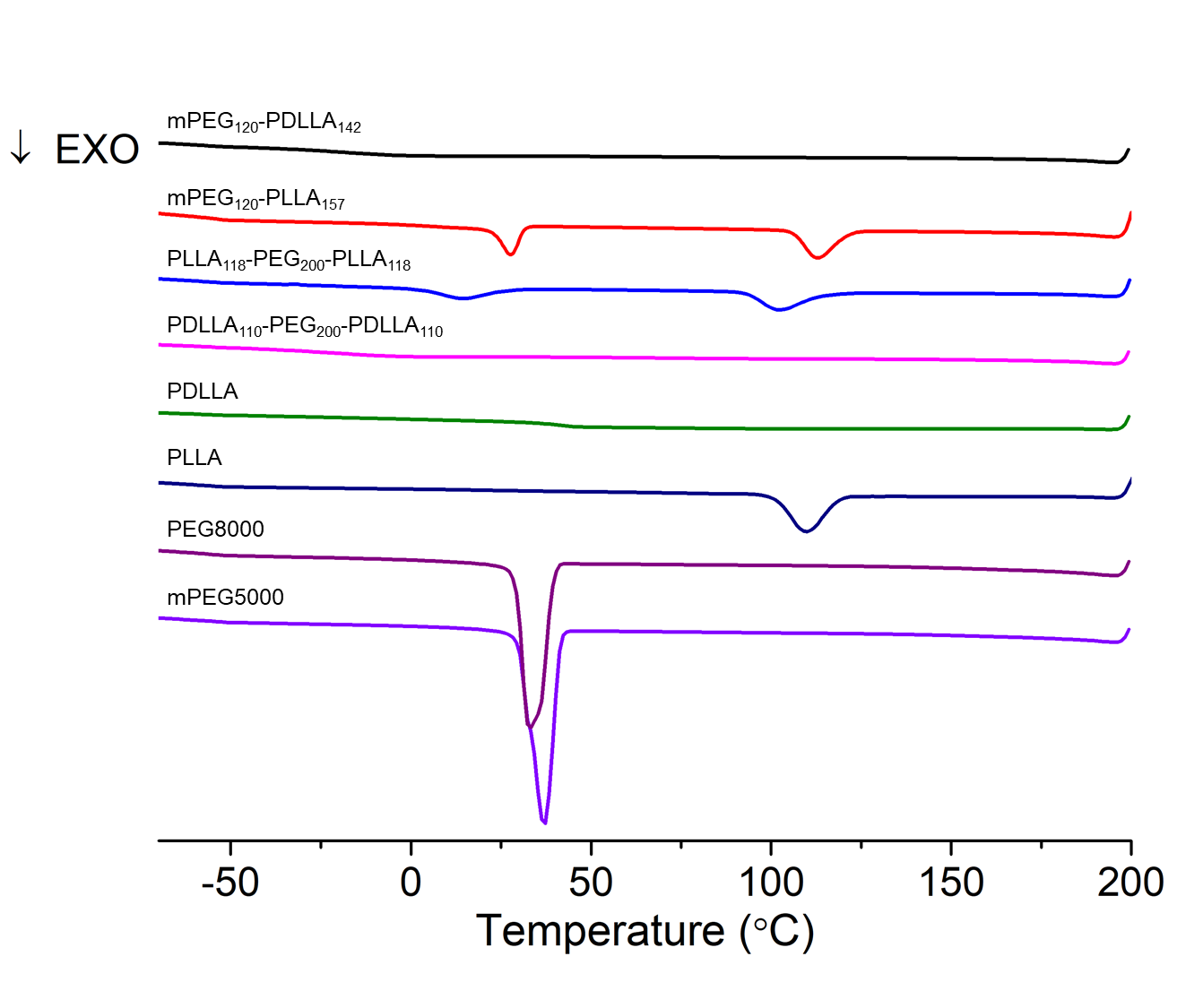


**FIGURE S9. First cooling curves of the polymers.** DSC thermograms of mPEG_120_-*b*-PDLLA_142_ (sample 2, black), mPEG_120_-*b*-PLLA_157_ (sample 6, red), PLLA_93_-*b*-PEG_200_-*b*-PLLA_93_ (sample 20, blue), PDLLA_110_-*b*-PEG_200_-*b*-PDLLA_110_ (sample 10, magenta), PDLLA (sample 24, green), PLLA (sample 25, navy), PEG_200_ (purple), and mPEG_120_ (violet).


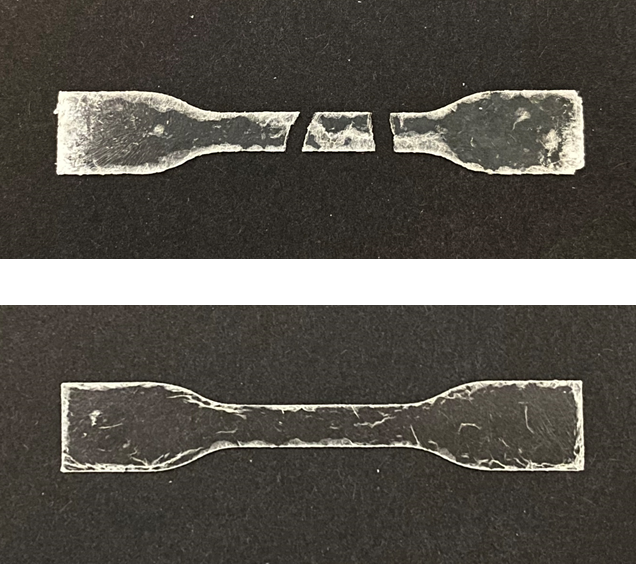


**A**

**B**

**FIGURE S10.** mPEG_120_-*b*-PLLA_261_ (sample 7) pressed (**A**) at 37 °C under 10 MPa for 48 h and (**B**) at 70 °C under 10 MPa for 48 h. The sample broke during handling and showed simple gluing of polymer particles at several places indicating not proper flow under pressure.


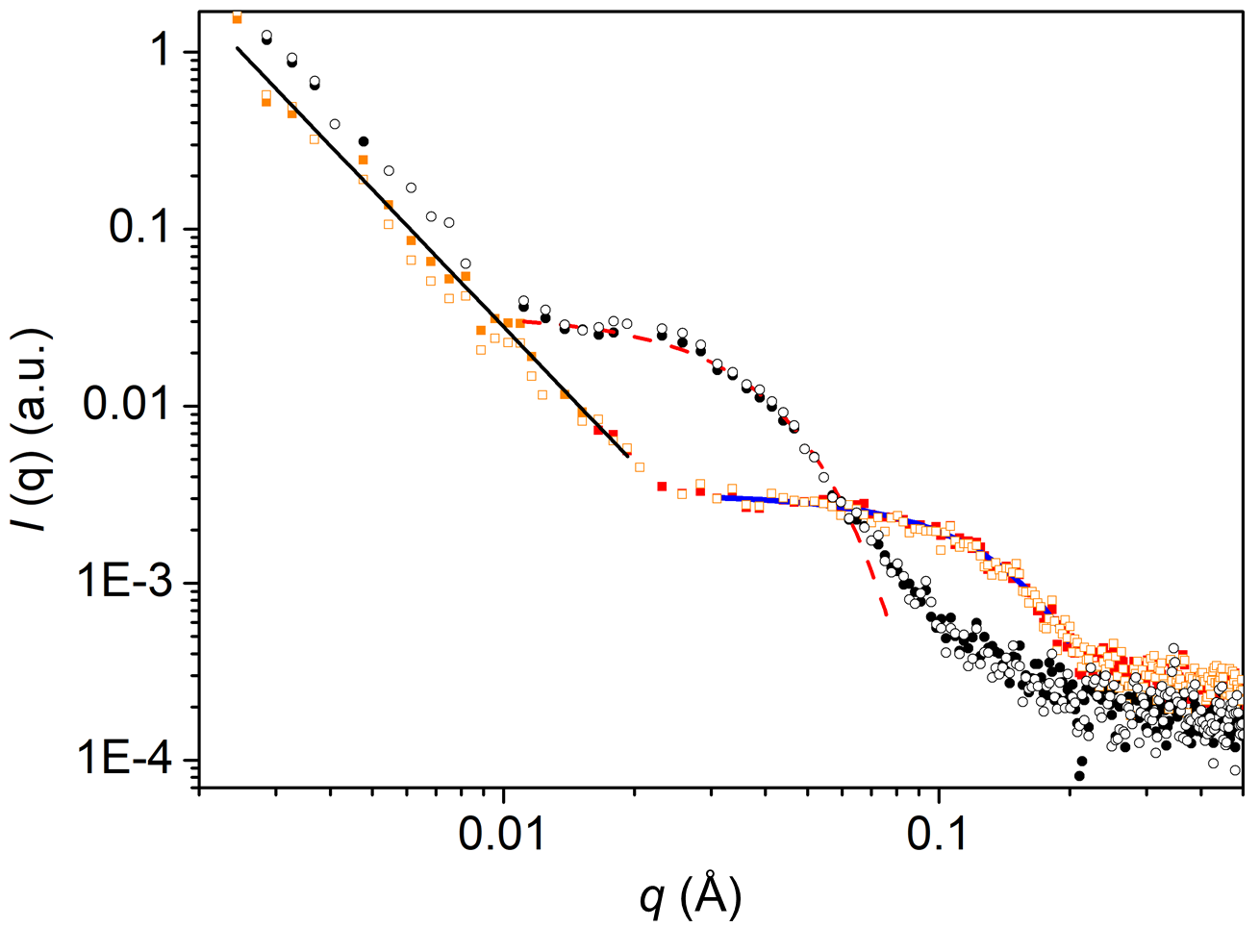


**FIGURE S11. SAXS data with beam at different directions relative to film plane.** SAXS data of PLLA_93_-*b*-PEG_200_-*b*-PLLA_93_ (sample 20) after pressing at different temperatures (black circles: film pressed at 135 °C under 10 MPa for 5 min, orange squares: film pressed at 37 °C under 10 MPa for 14 h). The data were taken with X-ray beam parallel (open symbols) or perpendicular (filled symbols) to the main film surface. The dashed lines correspond to Guinier fits, whereas the solid line is a *q^-3^* scaling law.

As consequence in a PLLA-*b*-PEG-*b*-PLLA triblock copolymer where PLLA and PEG are covalently bound, the phase segregation may change dramatically with temperature. Small angle X-ray scattering (SAXS) with X-ray beam parallel or perpendicular to the film surface was performed to investigate the possibility of block segregation in PLLA_93_-*b*-PEG_200_-*b*-PLLA_93_ films (see Fig. S11). The radial averaged intensities (1D SAXS) are independent from the beam-sample geometry. The scattering intensities show a ~*q*^-3^ power law at low *q* and a shoulder at intermediate *q* values. The *q*^-3^ scaling hints to the existence of a (maybe pore-like) structure in the nano- and mesoscale, whereas the shoulder is considered as characteristic for an immiscible block. In a first approximation the averaged size of these blocks may be described by Guinier’s law using a radius of gyration of *R_g_* = 1.2 nm for the film pressed at 37 °C and of *R_g_* = 4.5 nm for the film pressed at 135 °C. These values correspond to the radius of a sphere of *R* = 1.5 nm for film pressed at 37 °C and *R* = 5.8 for film pressed at 135 °C, respectively based on Equation (5):

|  | ${\text{R}_{\text{g}}}^{\text{2}}\text{=}\text{(3/5)}\text{R}^{\text{2}}$ | (5) |
| --- | --- | --- |

Therefore, we assume that indeed a phase separation took place, leading to significant smaller PEG aggregated in film pressed at 37 °C (compared to film pressed at 135 °C). Unfortunately, due to the low contrast and bad signal to noise ratio, the determination of the exact geometrical structure of the pre-aggregates is beyond scope, but the 2D SAXS obtained in parallel beam geometry points to an anisometric structure (exemplarily shown for film pressed at 37 °C and 135 °C in Fig. S12 and S13, where this feature is most pronounced).


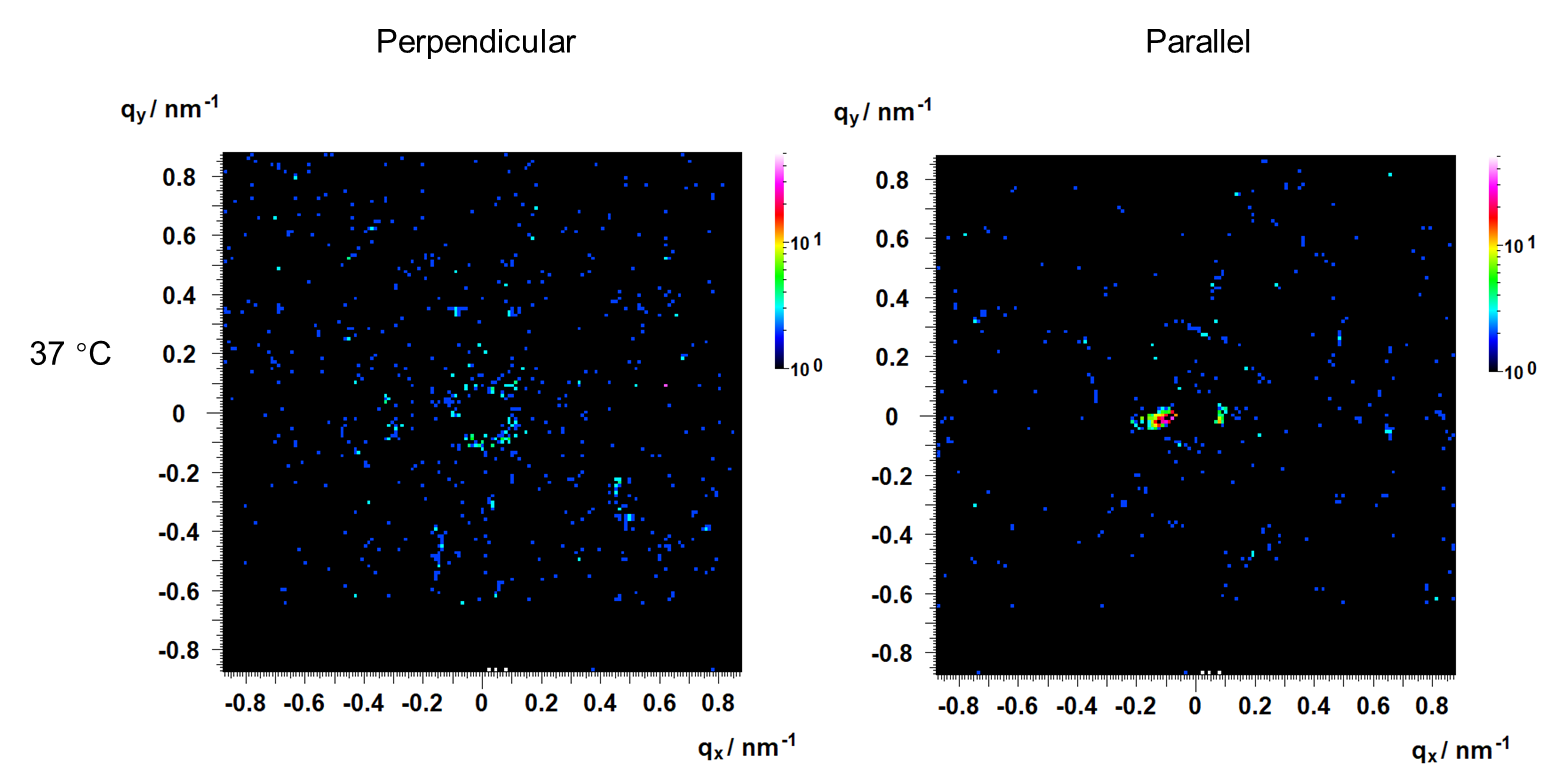


**FIGURE S12. 2D SAXS intensities with beam at different directions relative to film plane.** SAXS intensities of PLLA_93_-*b*-PEG_200_-*b*-PLLA_93_ (sample 20) film pressed at 37 °C under 10 MPa for 14 h. The data were taken with X-ray beam perpendicular (left, isotropic) and parallel (right, anisotropic) to the main film surface. The scattering contribution of air was subtracted. The differences between both pattern points to an anisotropic feature inside the sample, as it may for example result from worm-like or lamellar substructures inside the sample.


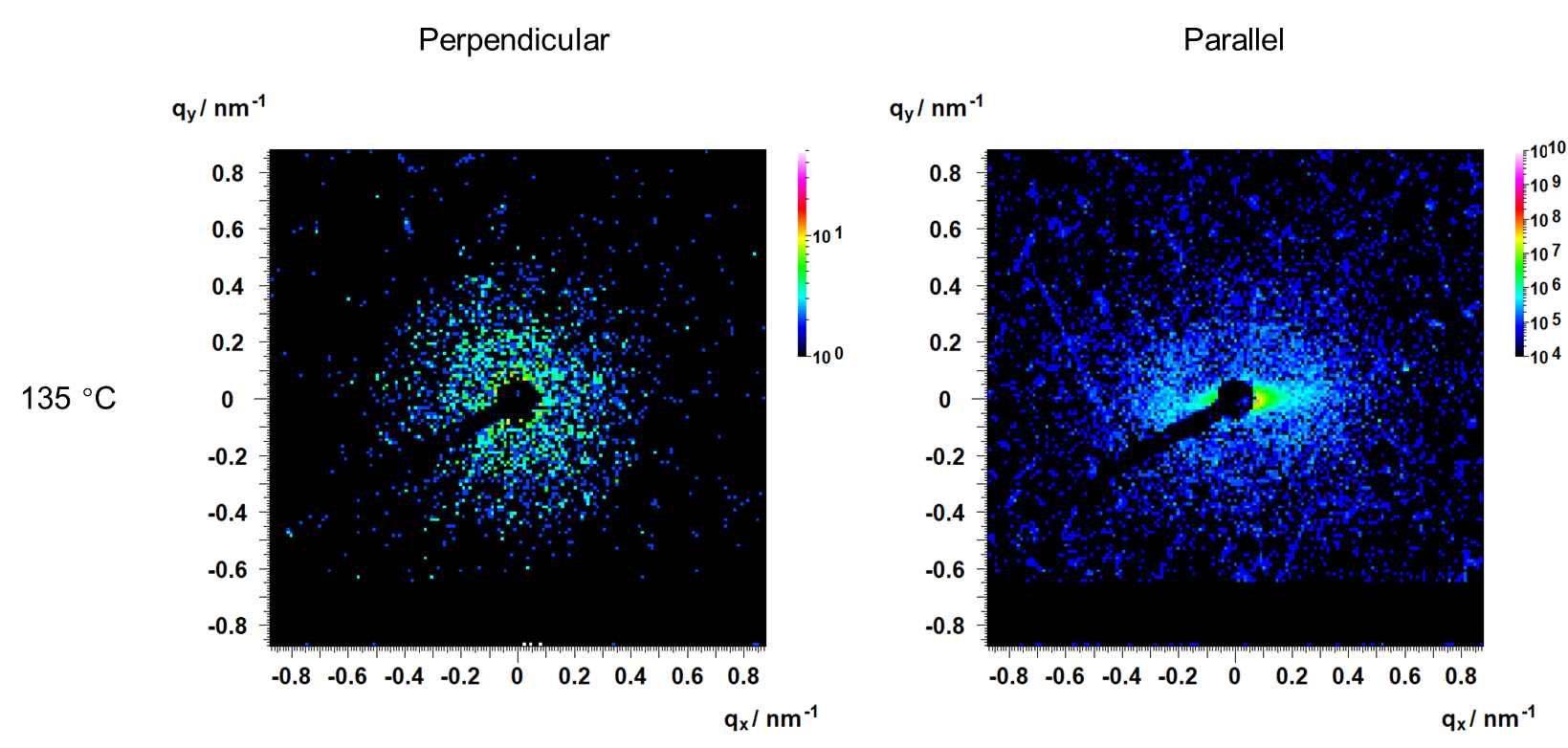


**FIGURE S13. 2D SAXS intensities with beam at different directions relative to film plane.** SAXS intensities of PLLA_93_-*b*-PEG_200_-*b*-PLLA_93_ (sample 20) film pressed at 135 °C under 10 MPa for 5 min. The data were taken with X-ray beam perpendicular (left, isotropic) and parallel (right, anisotropic) to the main film surface. The scattering contribution of air was subtracted. The differences between both pattern points to an anisotropic feature inside the sample, as it may for example result from worm-like or lamellar substructures inside the sample.

TEM result and discussion

For the sample pressed at 37 °C, TEM revealed (Figure 2D in main manuscript) irregular morphologies with disordered nanophase domains of sizes in the range of 5 to 15 nm. With the assumption that both blocks are equally crystallized the darker areas correspond to the PEG-rich phase and the brighter areas to the PLLA-rich phase as ethers are more readily stained by RuO_4_ than esters. In case of differences in crystallinity the phases cannot be assigned to PEG or PLLA since the staining process with RuO_4_ is diffusion-based and amorphous regions are more readily stained than crystalline regions. In addition, there are some areas showing irregular morphologies with larger disordered nanophase domains up to 100 nm in diameter as represented in Figure S14.

For the sample pressed at 135 °C, TEM revealed (Figure 2E in main manuscript) randomly oriented lamellar domains with lamellar crystal thicknesses of roughly 5 nm. The assignment of PEG or PLLA to the different phase based on greyscales is not possible since within the semicrystalline polymer the amorphous regions are more readily stained than the crystalline regions.


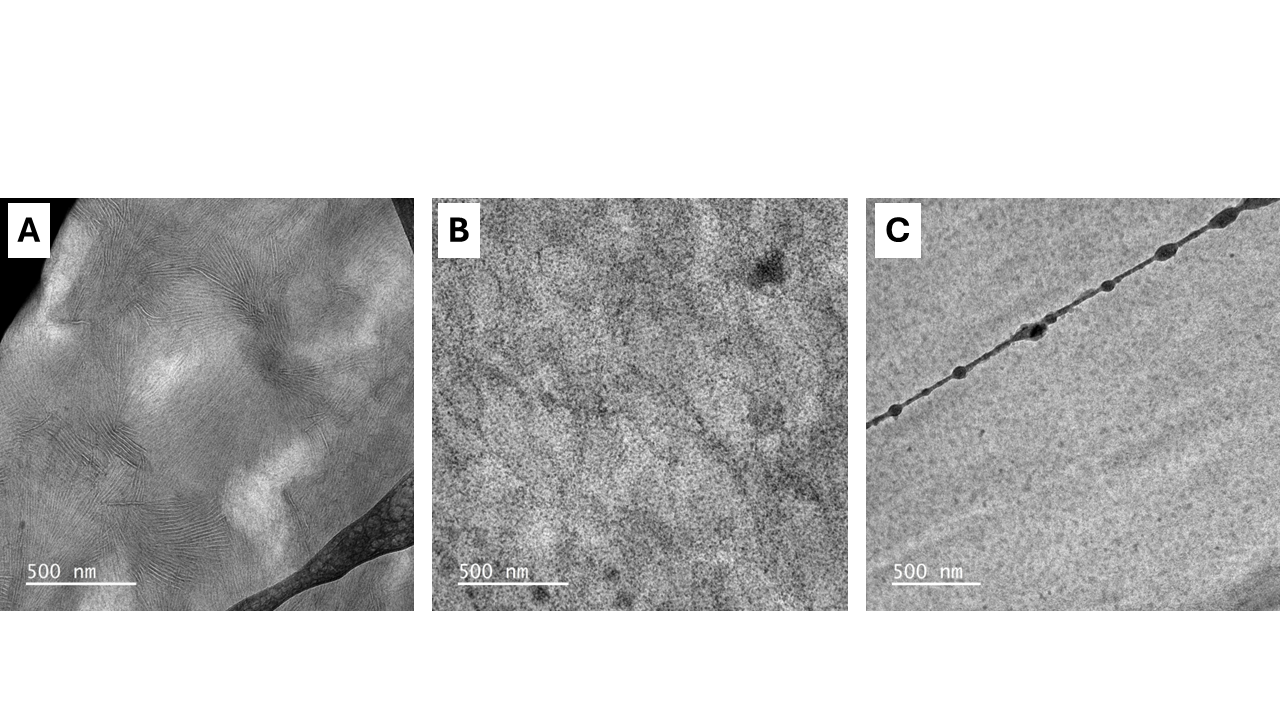


**FIGURE S14.** **TEM micrographs of RuO_4_-stained thin sections of PLLA_93_-*b*-PEG_200_-*b*-PLLA_93_** **sample.** (**A**) PLLA_93_-*b*-PEG_200_-*b*-PLLA_93_ pressed at 135 °C/10 MPa/5 min, which shows semicrystalline lamellar morphology. (**B**) and (**C**) PLLA_93_-*b*-PEG_200_-*b*-PLLA_93_ pressed at 37 °C/10 MPa/14 h, which shows different sizes of disordered nanophase domains.

Biodegradation results


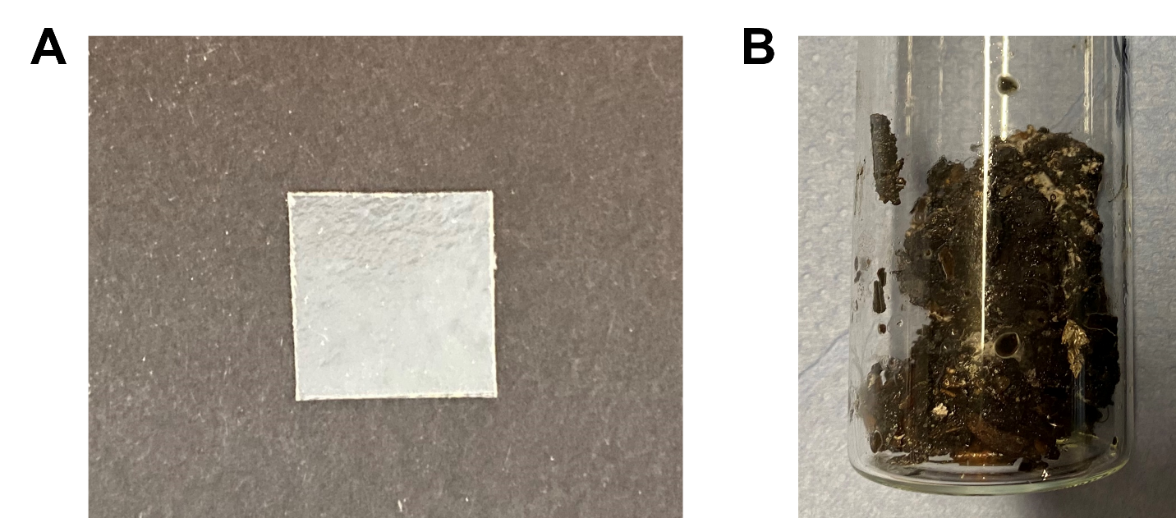


**FIGURE S15. Photographic images of PLLA_93_-*b*-PEG_200_-*b*-PLLA_93_ before and after biodegradation in compost.** (**A**) Pristine PLLA_93_-*b*-PEG_200_-*b*-PLLA_93_ film used for compost degradation. (B) Residual PLLA_93_-*b*-PEG_200_-*b*-PLLA_93_ sample after 60 days of compost degradation. The film was almost completely degraded, leaving only small residues after the biodegradation.

Environmental investigation

The cell viability was the lowest for the sample with the concentration of 100 µg/mL for Caco2 (87.9 % of control viability) and the highest for that of 1000 µg/mL in dTHP-1 (106.8 % of control viability). The increase in cell viability for the highest concentration in dTHP-1 could be explained by a stimulation of the cells and therefore higher metabolic activity, which consequently did not result in acute cytotoxicity. For the J774A.1 cells treated with all suspension concentrations, for the Caco2 cells with all suspension concentrations higher than 10 µg/mL, and for the dTHP-1 cells with suspension concentration of 0.1, 1 and 100 µg/mL, the viabilities were significantly lower than the untreated cells (Kruskal Wallis test and Games Howell post hoc testing: J774A.1: 0.1 = p < 0.01 & 1, 10, 100, 500, 1000 = p < 0.001; Caco2: 10, 100, 500, 1000 = p < 0.01; dTHP-1: 0.1 & 1 = p < 0.05, 100 = p < 0.001). However, there were no significant viability differences between the used suspensions with all concentrations (J774A.1 and Caco2) indicating no acute dose-dependent cytotoxicity of the PLLA-*b*-PEG-*b*-PLLA triblock copolymer particles in suspension with particle size ~300 nm. Overall, no severe acute cytotoxicity was observed for all cell lines and concentrations.

In the *Daphnia* acute toxicity test, no immobilization of *D. magna* was detected for any of the tested concentrations and time points. Therefore, no EC_50_ of the PLLA_118_-*b*-PEG_200_-*b*-PLLA_118_ (sample 21) can be given here up to a concentration of 350 µg/mL.

Chemical recycling of baroplastic

After chemical recycling in NaOH solution, the mixture was freeze-dried. After drying, the solid was again dissolved in water, and hydrochloric acid was used to neutralize the samples. The water was then removed by freeze-drying. Following removal of the aqueous phase, the solid product was extracted separately with chloroform and diethyl ether. For the chloroform extract, after solvent removal, the ^1^H NMR spectrum was recorded in CDCl₃, showing only a PEG signal (3.65 ppm). For the diethyl ether extract, after solvent removal, the ^1^H NMR spectrum was recorded in DMSO-d₆, showing signals from both lactic acid (1.25 ppm and 4.05 ppm) and PEG (3.35 ppm).


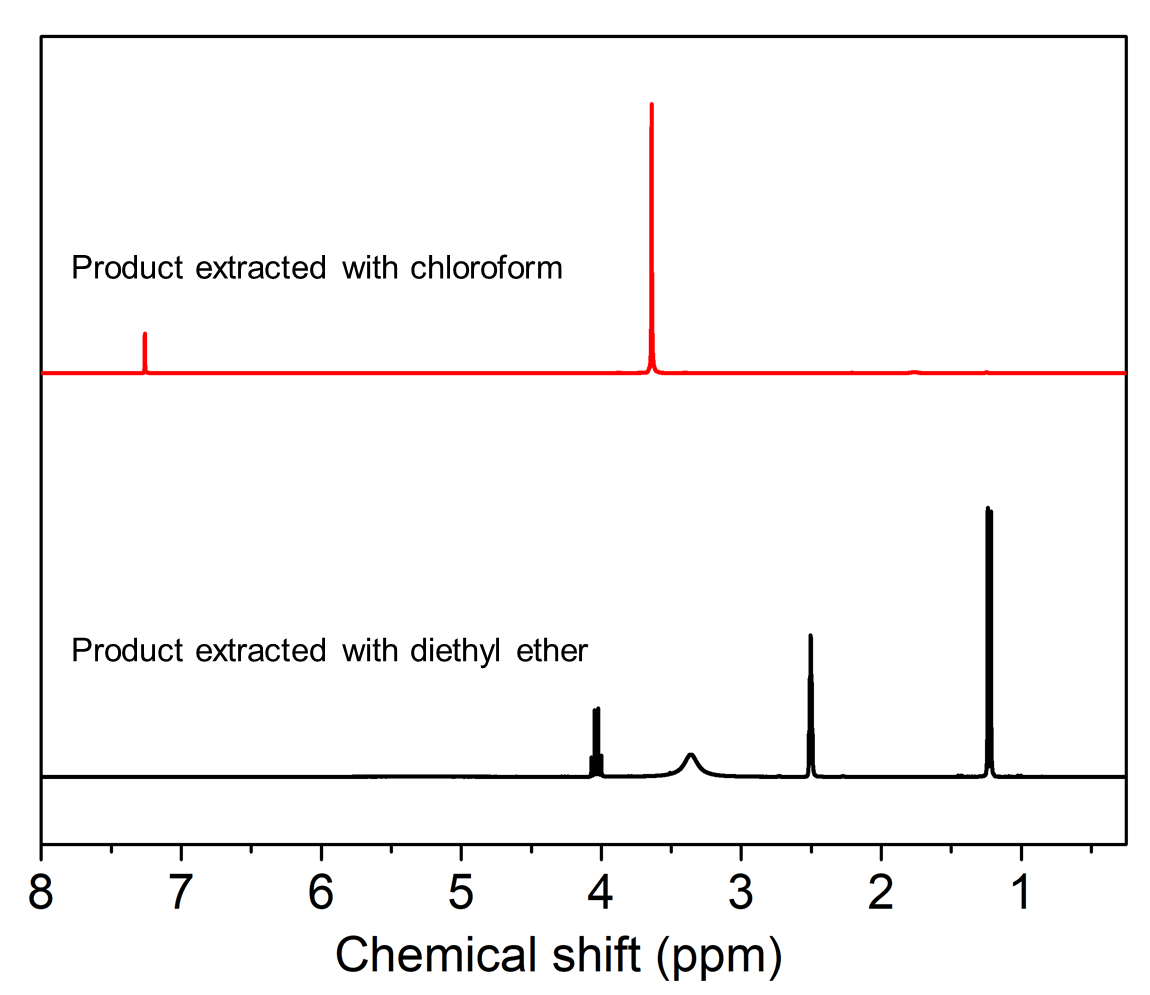


**FIGURE S16. ^1^H NMR spectra of the extractants from the hydrolytic degradation product of PLLA_93_-*b*-PEG_200_-*b*-PLLA_93_** **(sample 20 film).** The ^1^H NMR spectrum of the product extracted with chloroform (top) was recorded in CDCl₃ and shows only the PEG peak at 3.65 ppm. The ^1^H NMR spectrum of product extracted with diethyl ether (bottom) was recorded in DMSO-d_6_, showing both PEG and lactic acid peaks.

Protein encapsulation in baroplastic

The lifetimes extracted from the data in Figure S17 of the YPet protein immobilized in the triblock copolymer film are similar to the one of the native YPet powder (see Table S2). The decrease of the fluorescence intensity of the pressed film at 135 °C under 10 MPa for 5 min suggests that only a minority of the proteins remain intact and contributes to the signal.


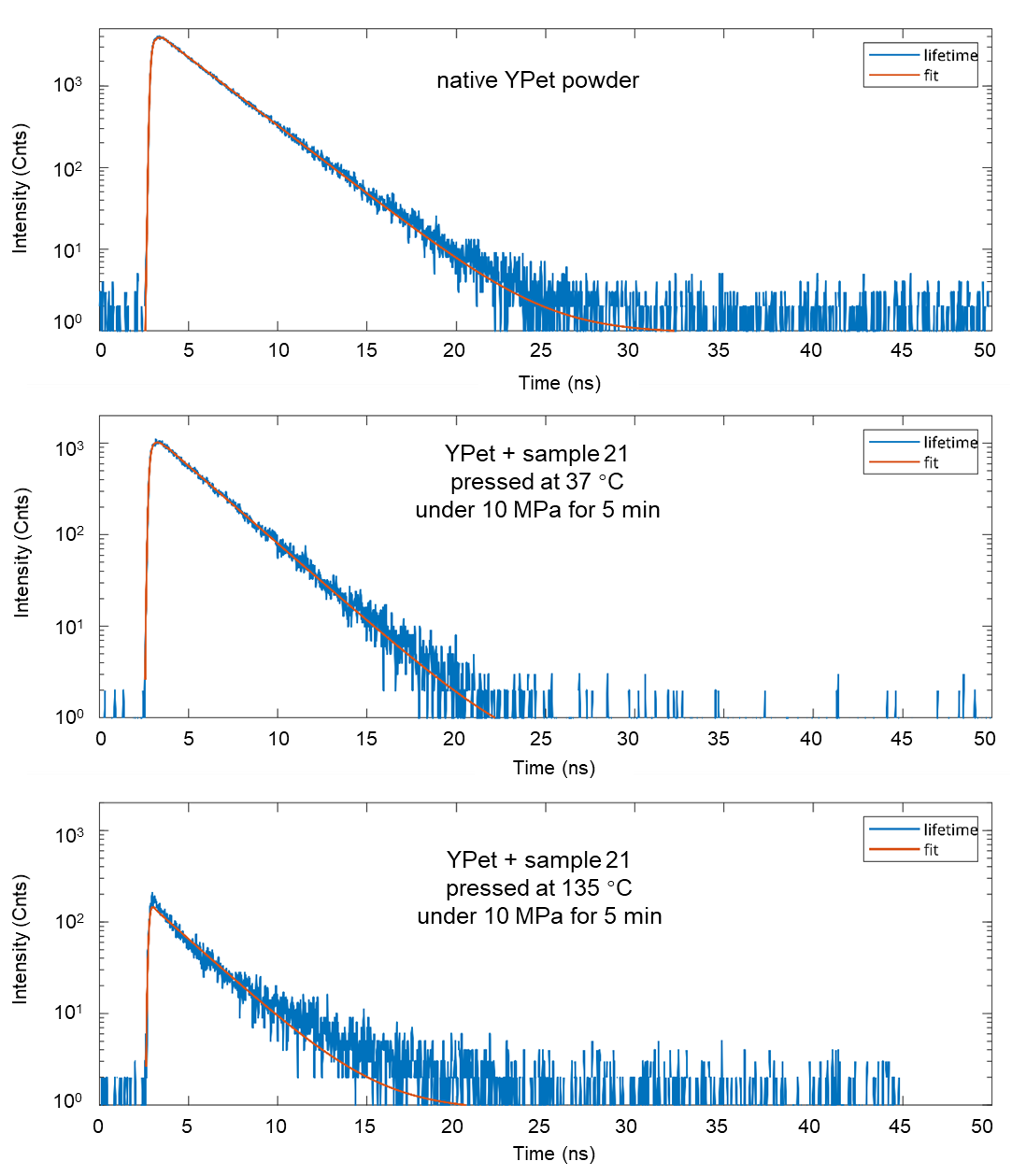


**C**

**B**

**A**

**FIGURE S17. YPet lifetime measurements.** (**A**) Lifetime histogram of native YPet powder. Lifetime histogram of YPet immobilized in PLLA_118_-*b*-PEG_200_-*b*-PLLA_118_ (sample 21) films pressed at (**B**) 37 °C under 10 MPa for 5 min, and (**C**) 135 °C under 10 MPa for 5 min. The intensity represents if YPet could kept its complete topological structure during processing.

**TABLE S2.** Fluorescence lifetime of native YPet powder and YPet immobilized PLLA_118_-*b*-PEG_200_-*b*-PLLA_118_ (sample 21) pressed film at 37 °C and at 135 °C under 10 MPa pressure for 5 min (ratio YPet : sample 21 = 99:1 (w:w)).

| Sample | Lifetime τ/ns |
| --- | --- |
| YPet powder | 2.61 ± 0.05 |
| YPet in sample 21 pressed at 37°C | 2.56 ± 0.05 |
| YPet in sample 21 pressed at 135°C | 2.48 ± 0.08 |


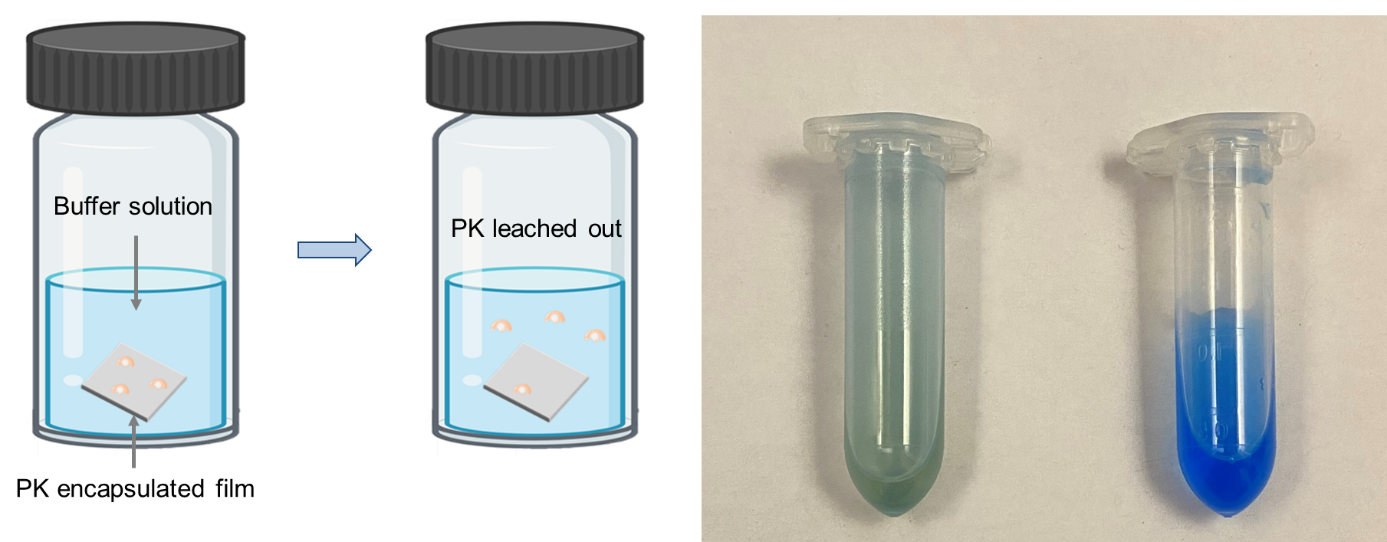


**A**

**B**

**FIGURE S18.** Activity test of the PK in PLLA_118_-*b*-PEG_200_-*b*-PLLA_118_ processed under baroplastic condition. (**A**) Schematic illustration. (**B**) Photograph of the Coomassie Brilliant Blue staining test with tris buffer (left) and with tris buffer / PK leachate (blue color refers to stained protein) which proves the activity of PK.


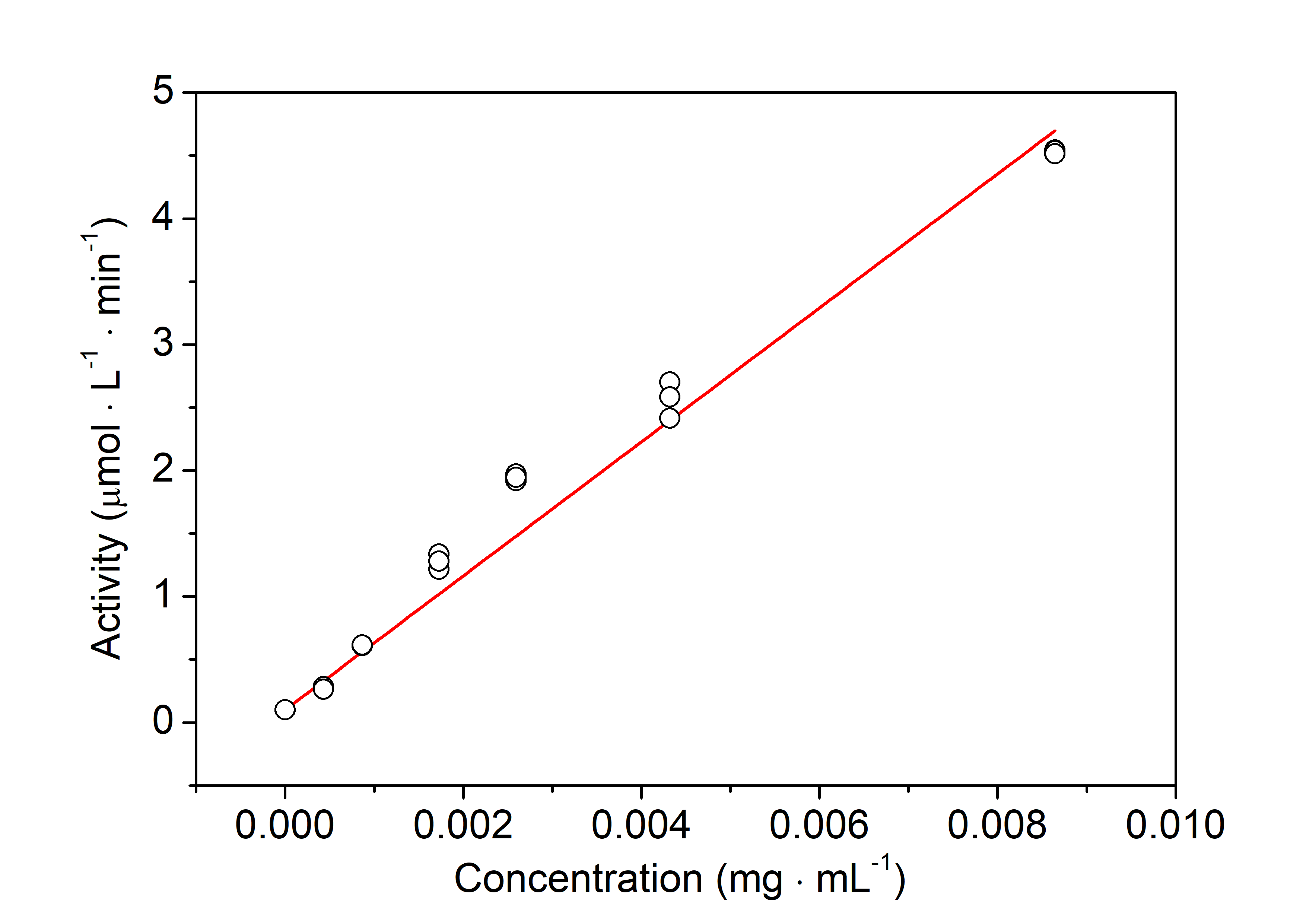


**FIGURE S19. Proteinase K activity measurement.** Plot of the calibration of proteinase K activity vs. concentration of proteinase K in buffer solution. Values of *n* =3 independent measurements are shown. The value was fitted by linear fitting using OriginLab 8 (y = ax+b, where a = 530 μmol/(g · min), b = 0 μmol/(L · min), R^2^ = 0.993). The function was used for the calculation of the concentration of proteinase K leachate from baroplastic film. The leachate of proteinase K is 0.3 mg/mL based on the calibration curve, which corresponds to 40% of active proteinase K leached out from sample 21 after 21 d. The leachate was diluted 100-fold before testing.
